# Synthesis of Polyimides, Polyamides, and Poly(Amide-Imides) in the “Green” Solvent N-Butyl-2-Pyrrolidone (TamiSolve NxG): Features, Optimization, and Versatility

**DOI:** 10.3390/ijms27031252

**Published:** 2026-01-27

**Authors:** Olesya N. Zabegaeva, Alexander V. Chuchalov, Dmitriy A. Khanin, Denis O. Ponkratov, Dmitriy A. Sapozhnikov

**Affiliations:** A.N. Nesmeyanov Institute of Organoelement Compounds, Russian Academy of Sciences, Vavilov Str. 28, Moscow 119334, Russia; zabegaeva@ineos.ac.ru (O.N.Z.); chuchalov@ineos.ac.ru (A.V.C.); d.a.khanin@gmail.com (D.A.K.); mattur@mail.ru (D.O.P.)

**Keywords:** N-butyl-2-pyrrolidone, TamiSolve NxG, polyimide, polyamide, poly(amide-imide), synthesis

## Abstract

Owing to their outstanding thermal and mechanical properties, polyimides (PIs), polyamides (PAs), and poly(amide-imides) (PAIs) are essential for developing and manufacturing modern high-tech products, including electroactive ones. Despite their large-scale production for diverse applications, the synthesis of these polymers traditionally relies on highly toxic solvents such as N,N-dimethylacetamide, N,N-dimethylformamide, N-methyl-2-pyrrolidone (NMP), and *m*-cresol. This work investigates the possibility of replacing these hazardous solvents with a more sustainable and “green” alternative, N-butyl-2-pyrrolidone (NBP). We have thoroughly studied and analyzed the synthesis of various PIs, PAs, and PAIs via one- and two-step polycondensation of tetracarboxylic acid dianhydrides with diamines, low-temperature polycondensation of terephthaloyl chloride with diamines, and low-temperature polycondensation of tetracarboxylic acid dianhydrides and terephthaloyl chloride with diamines, respectively. Our results demonstrate that substituting NBP for NMP presents distinct characteristics and outcomes for each process. By optimizing the reaction conditions, we were able to obtain high-molecular-weight products (M_n_ = 37–346 kDa; M_w_ = 133–537 kDa) for all polymer classes studied. Thus, this work establishes NBP as a suitable and promising solvent for synthesizing PIs, PAs, and PAIs with diverse chemical structures and tunable molecular weight characteristics.

## 1. Introduction

Polyimides (PIs), polyamides (PAs), and polyamide-imides (PAIs), as key classes of high-performance polymers, are of significant academic and commercial importance. Owing to their outstanding thermal and mechanical properties, along with the ability to tailor various characteristics, these polymers are among the most well-known and widely used. In addition to their widespread application in the (micro)electronics industry and aerospace engineering, they are also of considerable interest for producing highly thermostable coatings and filters for scrubbing harmful emissions [1,2,3,4,5,6], gas separation and pervaporation membranes [7,8,9,10,11,12,13], optical devices [10,13,14,15], flexible transparent films for displays and solar cells [16,17,18,19,20], and stimuli-responsive materials [10,13,21,22,23], and others [10,13,20]. Furthermore, due to the unique structural features of their macromolecules, these polymers are attractive for the application in electroactive devices [10,24,25,26].

Traditionally, the synthesis of PIs, PAs, and PAIs has relied on polar aprotic solvents such as N,N-dimethylacetamide (DMAA), N,N-dimethylformamide (DMF), or N-methyl-2-pyrrolidone (NMP). Unfortunately, these widespread solvents are reprotoxic and cause serious eye, skin, and respiratory irritation. Classified under REACH as “Substances of Very High Concern” and subject to authorization, they are prime candidates for substitution in the chemical industry [27,28,29]. Consequently, the need to adopt less hazardous and more environmentally benign solvents is becoming increasingly urgent and relevant.

In recent years, N-butyl-2-pyrrolidone (NBP), also known under the trademark TamiSolve NxG (Eastman Chemical Company), has been actively promoted as a “green” alternative to NMP, DMAA, and DMF [30,31,32,33]. This dipolar aprotic solvent, characterized by a high boiling point (241 °C), high chemical and thermal stability, aligns with several principles of Green Chemistry. Its advantages include lower toxicity and reduced human exposure hazards, a bio-based origin, and enhanced biodegradability [31,32,33,34,35,36]. Furthermore, an environmentally benign synthesis of NBP has been developed that eliminates the need for ammonia or alkylamines [37]. This method is based on Pd-catalyzed reductive N-alkylation and decarboxylation of renewable glutamic acid in an aqueous medium. NBP has been reported to serve as a highly effective substitute for NMP and other conventional dipolar aprotic solvents in the synthesis of various organic compounds [32,38,39,40,41].

It is known that NBP possesses a dissolving capacity similar to that of NMP, DMAA, and DMF, indicating its potential for preparing homogeneous polymer solutions [31,35,42,43]. For this reason, it has been extensively studied as a “green” solvent for producing membranes and films from PIs [28,33,44,45,46], fluoropolymers [33,42,47,48,49], poly(ether)sulfones [33,50,51], cardo polyetheretherketone [49,52], and other polymers [33,35,53]. Moreover, a mixture of NBP and di(propylene glycol) methyl ether (1:1 *w*/*w*) has been successfully applied for casting self-healable waterborne coatings as well [54]. These findings create favorable conditions for the broader adoption of NBP in the manufacture of various functional materials.

NBP has been thoroughly tested as a solvent for membrane fabrication. However, traditional polymer production methods still rely on toxic polar aprotic solvents that have significant negative impacts on the environment and human health. To date, only a limited number of publications have addressed polymer synthesis in NBP. The international patent [55] describes the use of various improved N-alkyl-2-pyrrolidone solvents for dissolving or forming a wide range of polymers, including cellulose derivatives, PAs, poly(amide/ester)imides, polyethers, polyesters, poly(ether)sulfones, and polyurethanes. Nevertheless, it presents only one example of polymer formation in NBP: a low-molecular-weight PAI (M_w_ = 8100 g/mol) obtained from trimellitic anhydride and 4,4′-methylenediphenyl diisocyanate. Furthermore, NBP/ionic liquid mixtures have been explored as alternatives to traditional solvents for synthesizing aromatic PAs [56]. Although the polymers formed in these mixtures generally exhibit lower molecular weights, this approach is promising for the in situ manufacture of high-strength fibers directly from the reaction solution. A European patent application is dedicated to the preparation of polyimides in N-C_3–5_ alkyl-2-pyrrolidone [57]. The reaction proceeds via a two-step method involving poly(amic acid) (PAA) formation followed by chemical or thermal imidization. While the patent notes the high viscosity of the resulting PAA intermediates, it provides no data on the final PIs. Although PIs can also be obtained via the direct one-step high-temperature polycondensation of dianhydrides and diamines [58,59], there is only one report mentioning the use of NBP as a reaction medium for this method [60]. The authors of that study compared the physical and gas-transport properties of the resulting PI membranes but did not provide a detailed analysis of the molecular weight characteristics of polymers obtained by different routes.

Despite successful attempts to utilize NBP as an alternative to traditional toxic solvents, the scarcity of data on polymer formation in this medium remains a significant challenge. Herein, we have studied the key features of PI, PA, and PAI synthesis in NBP as the reaction medium. Two distinct approaches to PI formation were investigated: one-step high-temperature polycondensation and the two-step process involving poly(amic acid) (PAA) intermediates. The effects of chemical and thermal cyclization of the PAA intermediate, as well as thermal imidization with azeotropic distillation, on the molecular weights of the polymers were examined. The versatility of NBP in one-step formulation of PIs with various structures was evaluated. PAs were obtained via low-temperature polycondensation of terephthaloyl chloride (TPC) with aromatic diamines. PAI synthesis was carried out from TPC, various tetracarboxylic acid dianhydrides, and diamines, using acetic anhydride and pyridine as the cyclization (imidization) mixture. The properties of the resulting PIs, PAs, and PAIs were systematically compared with those of polymers produced in conventional NMP.

## 2. Results and Discussion

### 2.1. Polyimide Synthesis

The most common methods of obtaining PIs are two- and, to a much lesser extent, one-step polycondensation of diamines and tetracarboxylic acid dianhydrides. Both of these approaches were compared using the example of a highly soluble cardo PI (ODPA-AFL) formed from 4,4′-oxydiphthalic anhydride (ODPA) and 9,9-*bis*(4-aminophenyl)fluorene (AFL). 

#### 2.1.1. Comparison of Two- and One-Step Methods

**Scheme 1:**
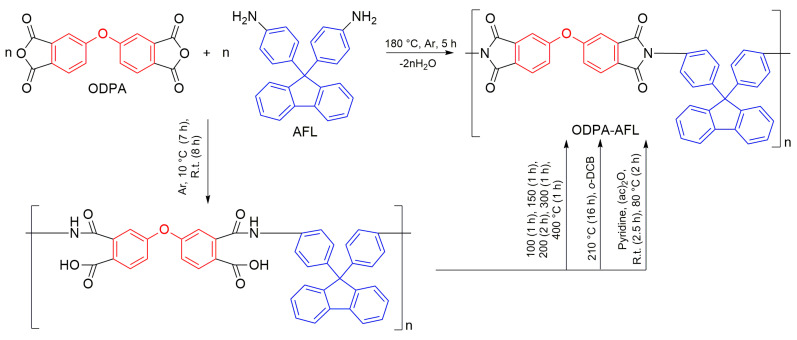
One- and two-step ODPA-AFL synthesis in NBP.

**Figure 1:**
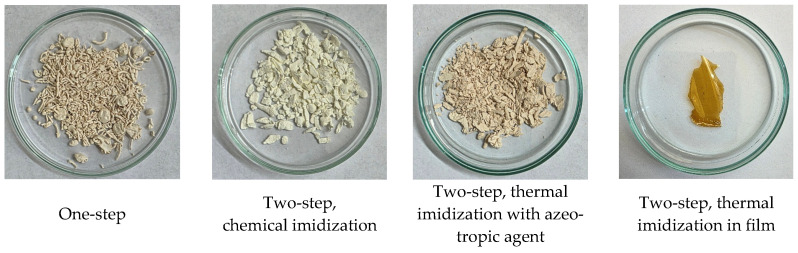
Photos of the ODPA-AFL samples obtained in NBP using various methods.

The synthesis of ODPA-AFL was realized out by a one-step method or by a two-step method through the formation of PAA intermediate and its subsequent imidization. The imidization of PAA in the second step was carried out chemically, thermally in a film and thermally in a solution with an azeotropic agent. The reaction routes and conditions, as well as the appearance of ODPA-AFL samples, are shown in Scheme 1 and Figure 1.

It is noteworthy that all the applied synthetic approaches for ODPA-AFL yielded a high-molecular-weight product. However, this could not be evaluated for thermally imidized ODPA-AFL film owing to its insolubility in NMP even when heated. The inherent viscosities (η_inh_) of the resulting soluble polymers fell within a narrow range of 0.7 to 0.8 dL/g, which is consistent with the η_inh_ of 0.8 dL/g measured for the PAA intermediate. Notably, the one-step high-temperature method produced high-molecular-weight ODPA-AFL within a shorter time and did not require the traditional acidic or basic catalysts. This method, along with the thermal imidization of PAA in solution using an azeotropic agent, enables the direct manufacture of various PI-based materials from the reaction mixture in a safer solvent environment.

As shown in Figure 1, the two-step approach employing chemical imidization yielded an almost colorless ODPA-AFL polymer. In contrast, both the one-step synthesis and the two-step method with thermal imidization produced colored samples. These observations are consistent with published data [18,60] and are important to consider when fabricating optically transparent films. The variation in color for polyimides of identical structure, formed by different routes, is attributed to side reactions occurring during thermal imidization at elevated temperatures. These reactions can lead to the formation of by-products and chromophoric groups within the polymer backbone, such as via the oxidation of terminal amino groups [18]. Chemical imidization, proceeding under milder conditions, minimizes such side reactions and thus yields a more colorless product.

We consider the one-step method to be the most economical and reproducible approach, offering precise control over the final polymer’s molecular weight. Consequently, this process was selected for more detailed investigation.

#### 2.1.2. Comparison of PI Synthesis in NMP and NBP

**Figure 2:**
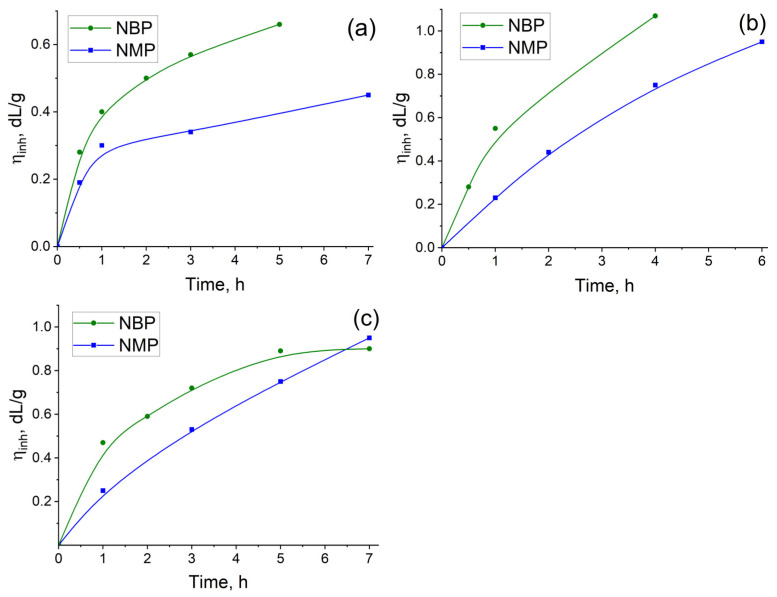
Dependences of η_inh_ for ODPA-AFL (**a**), ODPA-AFL_0.5_:DABA_0.5_ (**b**), and BTDA-AFL_0.5_:DABA_0.5_ (**c**) on the reaction time in NBP and NMP (T = 180 °C, Ar).

To evaluate the effect of replacing NMP with NBP in a one-step high-temperature polycondensation, the synthesis of three polyimides was studied in both solvents. These polymers were based on ODPA and 3,3′,4,4′-benzophenonetetracarboxylic dianhydride (BTDA) with the diamines AFL and 3,5-diaminobenzoic acid (DABA), due to the significant potential of cardo- and carboxylated PIs for manufacturing of a wide variety of functional materials [2,4,10,12,61]. Specifically, we compared the kinetics of inherent viscosity (η_inh_) increase—as an indicator of molecular weight growth—for ODPA-AFL, ODPA-AFL_0.5_:DABA_0.5_, and BTDA-AFL_0.5_:DABA_0.5_ during the reaction (Figure 2). Aliquots were taken from the reaction mixture at specific time intervals, and the η_inh_ of the isolated polymer was measured to monitor changes in molecular weight.

As evident from the kinetic curves presented in Figure 2, higher inherent viscosity values were achieved for all PIs during the initial hours of the reaction in NBP compared to the control syntheses conducted in NMP. Specifically, ODPA-AFL, ODPA-AFL_0.5_:DABA_0.5_, and BTDA-AFL_0.5_:DABA_0.5_ with high molecular weights (η_inh_ ≥ 0.4 dL/g) were formed in NBP after just one hour of reaction without any catalyst. In contrast, an equivalent viscosity (η_inh_ = 0.4 dL/g) for ODPA-AFL obtained in NMP was reached only after 7 h. These results clearly demonstrate a significant acceleration of the polycondensation process in the NBP medium. The incorporation of side carboxyl groups (as in DABA-containing copolymers) appears to catalyze this process further, which is consistent with published data [10,59]. The pronounced acceleration observed in NBP is likely attributable to its greater hydrophobicity compared to NMP. This property may facilitate more effective removal of water from the reaction mixture, thereby shifting the equilibrium towards polyimide formation. Although we have not found any information on this matter, it is possible that an azeotrope mixture of NBP and water may form, similar to the system of N-cyclohexyl-2-pyrrolidone and water [62]. Both hypotheses warrant further investigation. Nevertheless, the results obtained unequivocally demonstrate the efficacy of NBP as a reaction medium for production high-molecular-weight PIs within a significantly reduced timeframe. This opens promising avenues for optimizing polycondensation process parameters to meet specific application requirements.

#### 2.1.3. Versatility of NBP for the Synthesis of Different PIs

**Scheme 2:**
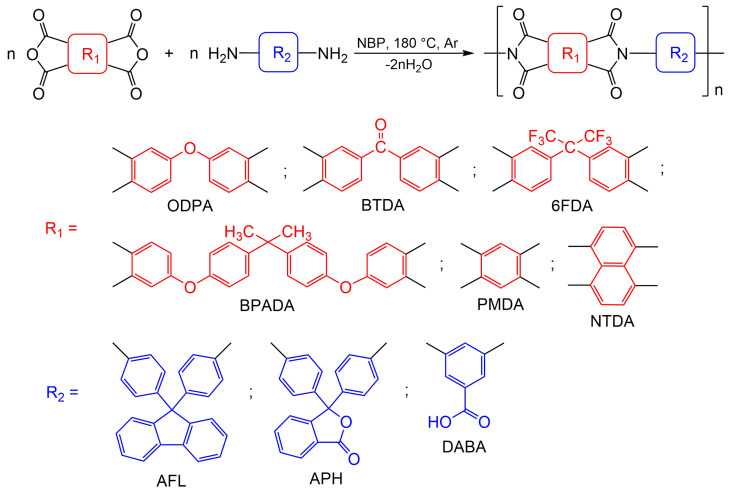
One-step high-temperature synthesis of PIs and the monomers used.

To evaluate the versatility of NBP for synthesizing various PIs—including those with flexible or rigid backbones and containing five- or six-membered imide cycles—we employed a series of dianhydrides: ODPA, BTDA, 4,4′-(hexafluoroisopropylidene)diphthalic anhydride (6FDA), 4,4′-(4,4′-isopropylidene diphenoxy)bis(phthalic anhydride) (BPADA), pyromellitic dianhydride (PMDA), and 1,4,5,8-naphthalenetetracarboxylic dianhydride (NTDA) (Scheme 2). This selection of tetracarboxylic acid dianhydrides was also guided by their significant potential for creating various functional (including electroactive) materials [10,18,21,25], as well as the need to investigate the synthesis of polymers from monomers with different inherent reactivities. The molecular weight characteristics of the resulting PIs, as determined by viscometry and gel permeation chromatography (GPC), are summarized in Table 1.

ODPA (E_a_ = 1.30 eV), BTDA (E_a_ = 1.55 eV), and 6FDA (E_a_ ≈ 1.4 eV) exhibit comparable reactivity [63]. As noted earlier, ODPA-AFL, ODPA-AFL_0.5_:DABA_0.5_, and BTDA-AFL_0.5_:DABA_0.5_ form rapidly and remain soluble throughout the reaction. As expected, 6FDA-DABA—which contains hexafluoroisopropylidene groups in the backbone—is also soluble in NBP and forms high-molecular-weight polymers (M_n_ = 275–346 kDa, M_w_ = 430–537 kDa) in both NBP and NMP (Table 1, Figures S17–S30). BPADA (E_a_ = 1.12 eV) [64] is the least reactive dianhydride in this series. Nevertheless, after 6 h of reaction in NBP, BPADA-DABA achieved an η_inh_ = 0.8 dL/g, M_n_ = 239 kDa, and M_w_ = 440 kDa. These results demonstrate that high-molecular-weight PIs can be obtained from less reactive monomers in NBP, even without catalytic additives.

Due to the ability of imide cycles to undergo reversible redox reactions and their increased stability, PMDA- and NTDA-based (poly)imides are actively considered for “Post-Lithium Energy Storage Applications” [25]. These dianhydrides contribute to the formation of PIs with the most rigid macromolecular backbones. It is well known that the highly reactive PMDA (E_a_ = 1.90 eV) [63] is also prone to side reactions. In our study, a highly viscous PMDA-AFL solution formed in NBP after 4 h of reaction, yielding the polymer with M_n_ = 191 kDa, M_w_ = 320 kDa, and η_inh_ = 0.9 dL/g. In contrast to PMDA, NTDA exhibits lower reactivity due to the high stability of its six-membered anhydride ring [65]. Nevertheless, high-molecular-weight PIs based on NTDA can be successfully obtained in NMP [59]. We observed that both NTDA-APH and NTDA-APH_0.5_:DABA_0.5_ precipitated during synthesis in NBP, leading to the formation of low-molecular-weight products (η_inh_ = 0.2–0.3 dL/g, M_n_ ≈ 12 kDa, M_w_ = 26–56 kDa) that remained soluble in NMP (Table 1). The insolubility of oligoimides based on NTDA can also be an additional technological advantage, for example, when there is no need to obtain high-molecular-weight compounds. The product is easily separated from the solvent, eliminating the use of a large amount of precipitant. Hansen parameters, particularly the dispersion component and the hydrogen bonding component, influence the solvent’s ability to stabilize or destabilize π-π interactions in aromatic systems. NBP has a lower capacity for specific interactions compared to NMP, which may promote chain aggregation via π-π stacking, leading to a locally high polymer concentration and precipitation before reaching high molecular weight [28].

It is noteworthy that the inherent viscosity and molecular weight values for all PIs are internally consistent. The obtained PIs are soluble in conventional polar aprotic solvents such as NMP, DMAA, DMF, and DMSO. BPADA-DABA and 6FDA-DABA exhibit solubility in cyclohexanone as well.

The structure of PIs has been confirmed by FT-IR spectroscopy (Supporting Information, Figures S1–S10). All the polymer spectra contain characteristic bands: 1775–1785 cm^−1^ (asymmetric) and 1716–1725 cm^−1^ (symmetric)—C=O stretching bands in the imide (C=O stretching in acid overlaps with symmetric signals); 1352–1365, 1079–1099, and 719–756 cm^−1^—C–N stretching bands; 2700–3600 cm^−1^—O–H groups of DABA (generally indistinguishable). The signals in the IR spectra of polyimides based on NTDA are shifted to the lower frequency region.

A clear correlation exists between the chemical structure of the polyimides and their thermal properties (Table 1). Cardo homopolyimides ODPA-AFL exhibit the highest glass transition temperatures (T_g_ ≥ 350 °C). It was not observed for PMDA-APH until the beginning of decomposition, and its definition made no sense for NTDA-APH and NTDA-APH_0.5_:DABA_0.5_ due to their low molecular weights. The lower thermo-oxidative stability of PMDA-APH compared to ODPA-AFL is attributed to the degradation of its lactone rings [66]. As shown in Table 1, incorporating DABA fragments into the polymer chains slightly reduces the thermal stability of the corresponding PIs: the glass transition temperatures decrease due to meta-substitution in the backbone, and the temperatures of 10% weight loss (T_10%_) are lowered by initial decarboxylation. These trends agree well with previously published data [10,59]. As expected, BPADA-DABA has the lowest T_g_ (260 °C), a result of the increased chain flexibility imparted by its isopropylidene and ether bridging groups.

We have demonstrated that NBP is a suitable solvent for synthesizing a wide range of polyimides with variable chain rigidity, thermal stability, and functionality (side trifluoromethyl, cardo, and carboxyl groups), including those derived from low-reactivity dianhydrides such as BPADA. However, it is not effective for obtaining high-molecular-weight PIs containing six-membered imide rings, such as those derived from NTDA.

### 2.2. Polyamide Synthesis

**Scheme 3:**
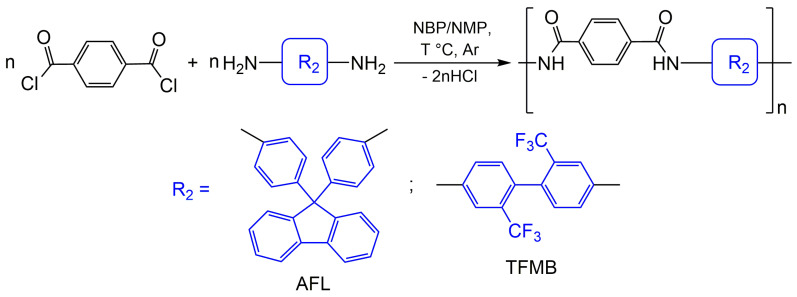
Synthesis of PAs by low-temperature polycondensation of TPC with diamine.

PAs were synthesized via low-temperature polycondensation of TPC with AFL or TFMB under identical conditions, except for the reaction temperature, which was maintained at −30 °C in NBP and −25 °C in NMP (Scheme 3). We observed that conducting the synthesis of TPC-AFL in NBP within the temperature range of −25 to −20 °C yielded polymers with lower molecular weights (η_inh_ ~ 0.4 dL/g) compared to those obtained at the optimal −30 °C (η_inh_ = 0.9 dL/g). Notably, the reaction mixtures in NBP turned yellow at temperatures of −25 °C or higher. Similar coloration was observed for analogous reactions in NMP, but at higher temperatures. This difference may be attributed to the occurrence of side reactions, as TPC remains soluble in NBP at −25 °C, whereas it is not soluble in NMP at this temperature.

The highly reactive chloroanhydride groups can form adducts with amide-type solvents [67]. To minimize such side processes, the synthesis should be conducted under heterogeneous conditions.

The polycondensation of aromatic diamines with dichloroanhydrides releases HCl. The free HCl can react with the NH_2_-groups, converting them into inactive ammonium salts. This is typically prevented by using organic bases to scavenge HCl. Given that NBP is a structural analog of NMP, it, like NMP, can also form stable salts with HCl, thereby acting as an internal acid acceptor [5].

**Figure 3:**
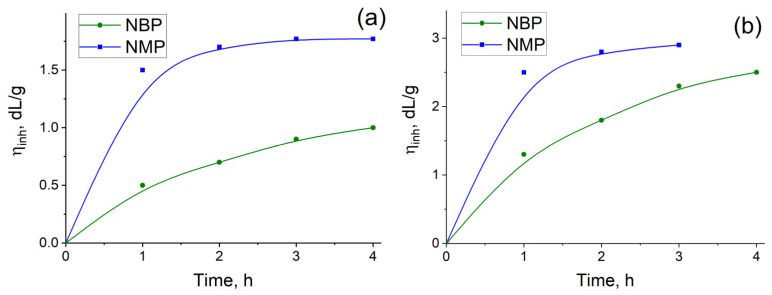
Dependence of the inherent viscosity of TPC-AFL (**a**) and TPC-TFMB (**b**) on the duration of the reaction in NBP and NMP.

The solvent nature significantly influences both the growth kinetics and the limiting η_inh_ of the resulting PAs. In all cases, the choice of NBP as the reaction medium leads to the formation of high-molecular-weight polymers, albeit over a longer time compared to the reference syntheses in NMP. As shown in Figure 3, the η_inh_ of TPC-TFMB and TPC-AFL formed in NMP reaches a plateau after 1.5–2 h, whereas a monotonic increase in η_inh_ is observed over 4 h when the polycondensation is conducted in NBP. The benchmark reaction using NMP yielded PAs with η_inh_ = 3.0 dL/g (TPC-TFMB) and η_inh_ = 1.8 dL/g (TPC-AFL) (Table 2). At the same time, the maximum inherent viscosity of isolated TPC-TFMB and TPC-AFL obtained in NBP were slightly lower—2.3 and 0.9 dL/g, respectively.

A small excess of TPC (3 mol.%) reduces the molecular weight of the resulting polymer, suggesting its potential involvement in side reactions with the solvent. A further increase in TPC loading (7 mol.%) leads to an even more pronounced decrease in the molecular weight of TPC-AFL (Table 2). This behavior indicates that the chloroanhydride groups likely terminate the growing polymer chains, thereby inhibiting further polymerization. A similar trend of reduced PA molecular weight upon replacing NMP with NBP has been reported previously [56]. The authors of that study suggested [56] that this effect could be due to local overheating caused by the exothermic reaction between TPC and the amino groups, which in turn promotes side reactions.

Here, to minimize the risk of local overheating, solid TPC was added gradually. The rate of the main reaction and the molecular weight of the resulting polymer, obtained via low-temperature polycondensation of diamines with dicarboxylic acid dichlorides, are likely influenced by the specific properties of the solvent used, such as its polarity, hydrogen-bonding capacity, and basicity [68]. Kinetic studies using the Menschutkin reaction as a model have demonstrated that reaction rates in dipolar aprotic solvents correlate strongly with the Kamlet–Taft solvatochromic parameter π* values correspond to faster reaction rates [69]. The π* value of NBP (0.77) is lower than that of NMP (0.92) [69], which aligns with the experimentally observed lower reaction rate in NBP in our study. Similar conclusions were drawn in [58], where it was shown that high-molecular-weight PAA form more readily in solvents with higher polarity. Conversely, the achievable molecular weight may also be related to the solvent’s dielectric constant (ε) [68]. For instance, during the low-temperature reaction of bis-(4-oxy-3-chlorophenyl-2,2-propane) with TPC, a tendency toward lower molecular weight of the resulting polyester was observed when using a solvent with a higher dielectric constant [68]. This trend is consistent with our findings for PA synthesis: the dielectric constants for NBP and NMP are 40 [70] and 32.2 [69], respectively, with the higher ε of NBP potentially contributing to the observed lower molecular weights compared to NMP.

The polymers obtained in NBP were isolated as white fibers (TPC-AFL) or hard yellowish pellets (TPC-TFMB). In contrast, both PAs synthesized in NMP were obtained as white fibers. The solubility of the PAs was tested in NMP, NBP, DMAA, DMF, and DMSO at ambient temperature and at 60 °C (Table 2). TPC-AFL samples, whether derived from NBP or NMP, exhibited good solubility in the listed amide solvents but were insoluble in DMSO. DMSO also proved unsuitable for dissolving the fluorinated TPC-TFMB polymers. Furthermore, the TPC-TFMB pellets isolated from NBP were difficult to dissolve in all tested amide solvents, even upon heating. In contrast, the reference TPC-TFMB fibers synthesized in NMP dissolved readily in these solvents at room temperature.

The GPC results are consistent with the η_inh_ data, as summarized in Table 2. PAs obtained in NBP exhibit slightly lower molecular weights than the benchmark polymers, with M_w_ ranging from 137 to 164 kDa and M_n_ from 46 to 51 kDa (Figures S31–S34). It is noteworthy that the molecular weights of TPC-TFMB, determined in both solvents, were lower than those of the cardo-based polyamide (TPC-AFL), despite its higher η_inh_ values. This apparent discrepancy may be attributed to the formation of strong hydrogen bonds in TPC-TFMB, which can lead to an overestimation of its molecular weight by viscometry.

The structure of PAs was confirmed by FT-IR spectroscopy and was found to be identical to that of the reference samples obtained in NMP (Supporting Information, Figures S11 and S12). All IR spectra exhibit characteristic absorption bands: the Amide I band (C=O stretch) at ~1660 cm^−1^, the Amide II band (N–H bend coupled with C–N stretch) at ~1515 cm^−1^, and a broad N–H stretching band in the 3300–3500 cm^−1^ region. It should be noted that for TPC-AFL, the Amide II signal is overlapped by a strong single band at 1508 cm^−1^, which corresponds to the *v*(C=C) vibration of the cardo moiety.

The thermal properties of PAs were evaluated by TMA and TGA as well (Table 2). The T_g_ values were 380 °C for TPC-AFL and 260 °C for TPC-TFMB. The thermo-oxidative stability of the polymers was found to be independent of the nature of the diamine and the solvent used for synthesis. T_10%_ for cardo-based and fluorinated PAs range from 470 (480) to 500 °C.

### 2.3. Poly(Amide-Imide) Synthesis

**Scheme 4:**
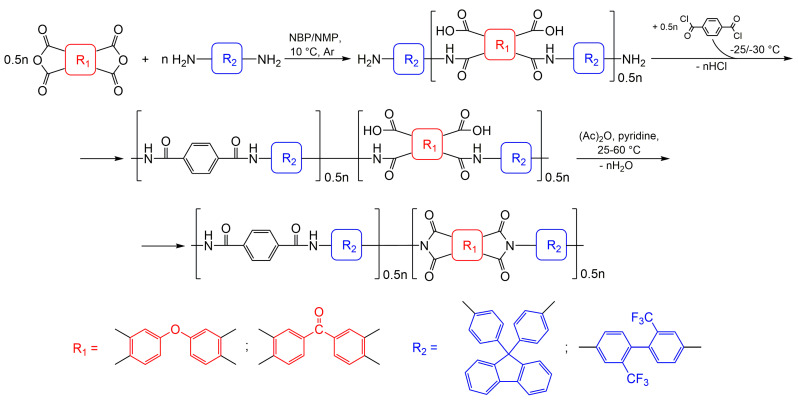
Synthesis of PAIs.

PAIs bearing cardo or trifluoromethyl groups were synthesized via low-temperature polycondensation of AFL or TFMB with aromatic tetracarboxylic dianhydrides and TPC, proceeding through poly(amide-amic acid) (PAAA) intermediates. The established synthetic protocol—including the sequence of reagent addition, their concentration, temperature, and duration—was based on a previously reported procedure using conventional NMP as the solvent [5]. The reaction route is presented in Scheme 4.

In the first stage, an amic acid intermediate was synthesized by adding the solid dianhydride to a cooled (10 °C) diamine solution. Subsequently, TPC was introduced portionwise, and the inherent viscosity of the resulting poly(amide-amic acid) (PAAA) was monitored. Cyclization of the PAAA was then carried out using a mixture of acetic anhydride and pyridine. Previous studies have shown that the temperature of the reaction between the intermediate amic acid and TPC significantly influences the molecular weight of both the PAAA and the final PAI [5]. Specifically, higher-molecular-weight PAIs form in NMP at −25 °C [5]. In the present study, using the synthesis of ODPA:TPC-AFL as a model, we investigated the effect of this reaction temperature on the viscosity of the final PAI. The reaction was performed in anhydrous NBP at temperatures of −20, −25, and −30 °C. As anticipated, the polymer with the highest molecular weight (η_inh_ = 0.6 dL/g) formed at −30 °C. Synthesis at −25 °C and −20 °C yielded PAIs with η_inh_ values two or more times lower—0.4 and 0.2 dL/g, respectively. Similarly to the PA synthesis described earlier, the reaction mixture acquired a yellowish coloration at the higher temperatures.

**Figure 4:**
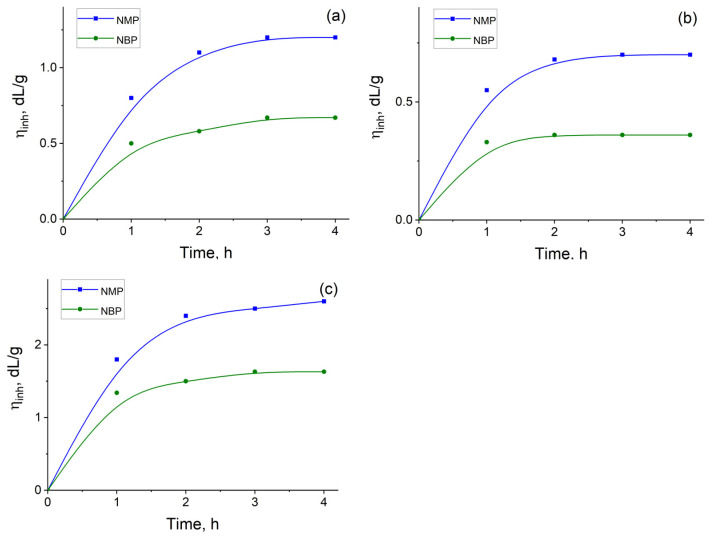
Dependence of the inherent viscosity of the PAAAs (ODPA:TPC-AFL (**a**), BTDA:TPC-AFL (**b**), and ODPA:TPC-TFMB (**c**)) on the duration of the reaction in NBP and NMP.

As shown in Figure 4, substituting NBP for NMP results in a significant decrease in the η_inh_ of PAAAs. Unlike the synthesis of PAs in NBP, the η_inh_ values for PAAAs show little dependence on the duration of the second reaction stage. In all cases, irrespective of the solvent used, the maximum molecular weight—as inferred from η_inh_—was attained after 1.5–2 h of reaction.

Consequently, the inherent viscosities of PAAAs synthesized in NBP were 0.4–0.7 dL/g (for cardo-based polymers) and 1.4–1.6 dL/g (for fluorinated polymers). In contrast, the benchmark PAAAs obtained in NMP exhibited η_inh_ values of 0.7–1.2 dL/g and 2.0–2.6 dL/g, respectively (Table 3). As summarized in Table 3, the inherent viscosities of the final PAIs were either comparable to or lower than those of the corresponding PAAA intermediates.

It was found that exceeding the equimolar concentration of TPC by 3, 7, and 10 mol.% increases the molecular weight of both the PAAA intermediates and the resulting PAIs during synthesis in NBP (Table 4). However, a 3 mol.% excess of TPC has only a minor effect on the molecular weight of the final PAIs.

The optimal results were achieved with a 7 mol.% excess of TPC. Under these conditions, the inherent viscosities of the isolated ODPA:TPC-AFL and BTDA:TPC-AFL polymers were 0.9 dL/g and 0.7 dL/g, respectively—significantly higher than the values of 0.6 dL/g and 0.3 dL/g obtained at equimolar loading (Table 4). A further increase in TPC content to 10 mol.% did not lead to additional gains in molecular weight. It is noteworthy that, in contrast, varying the TPC concentration in the comparative syntheses carried out in NMP had no significant effect on the inherent viscosity of the final PAIs.

High-molecular-weight polymers are essential for achieving superior performance in derived products such as films, coatings, and membranes. Therefore, the cardo-based PAIs formed in NBP with a 7 mol.% excess of TPC, which exhibited optimal molecular weights, were selected for further investigation. In contrast, fluorinated PAIs obtained under equimolar monomer loading already possess inherently high viscosities, which could limit their solubility in common solvents. Consequently, targeting even higher molecular weights for this class of polymers is not practical.

Most of the synthesized PAIs (from both solvents) were isolated as white or pale yellow fibers with good solubility in common dipolar aprotic solvents, including NMP, NBP, DMAA, DMF, and DMSO, at ambient temperature or upon heating (Table 5). However, when NBP was used for the synthesis of BTDA:TPC-TFMB, it yielded hard yellowish pellets that were soluble only in hot organic solvents. This contrasts with the readily soluble white fibers of the same polymer obtained from NMP.

The molecular weights of PAIs estimated from GPC data are in good agreement with their η_inh_ values (Table 5).

Overall, the polymers synthesized in NBP exhibit comparable or slightly lower molecular weights than their counterparts isolated from NMP. A notable exception is BTDA:TPC-AFL, which displayed an η_inh_ = 0.7 dL/g—exceeding that of the reference PAI from NMP (η_inh_ = 0.6 dL/g, even with a 7 mol.% excess of TPC; see Table 4). These results demonstrate that NBP is a suitable medium for synthesizing PAIs with high molecular weight characteristics, yielding polymers with Mn = 46–202 kDa, Mw = 195–384 kDa, and a polydispersity ranging from 1.9 to 4.2 (Figures S35–S42).

The structure of PAIs was confirmed by FT-IR spectroscopy (Supporting Information, Figures S13–S16). All PAIs exhibited characteristic absorption bands corresponding to their functional groups: asymmetric and symmetric imide C=O stretching at ~1780 and ~1723 cm^−1^, respectively; the Amide I band at ~1669 cm^−1^; the Amide II band at ~1523 cm^−1^; imide C–N–C stretching at ~1370 cm^−1^; and C–F stretching (from CF_3_ groups) in the 1248–1054 cm^−1^ region, with distinct bands at ~1248 and ~1173 cm^−1^. The absence of a broad absorption band in the 2600–3600 cm^−1^ region, which would correspond to O–H stretching vibrations of carboxylic acid groups, confirms the formation of a fully imidized structure [71].

As with the other polymer classes studied, the choice of reaction medium (NBP vs. NMP) does not significantly affect the T_g_ of the resulting PAIs. For cardo-based PAIs, T_g_ values were in the range of 370–380 °C, whereas the fluorinated PAIs exhibited lower T_g_ values of approximately 260–285 °C. The observed T_g_ values and their dependence on the PAI structure are consistent with previously published data [5,72].

The temperatures of 10% weight loss were determined from the original TGA thermograms and are summarized in Table 5. All PAIs exhibit high thermo-oxidative stability, which is independent of the reaction medium used for their synthesis. The T_10%_ values for these polymers range from 490 to 525 °C.

## 3. Materials and Methods

### 3.1. Materials

Monomers and other chemicals were purchased from TCI or Acros Organics. 2,2′-Bis(trifluoromethyl)benzidine (>98%, M_p_ = 188 °C), 9,9-bis(4′-aminophenyl)fluorene (>98%, M_p_ = 236–237 °C), 3,5-diaminobenzoic acid (98%, M_p_ = 236–237 °C), 3,3′,4,4′-diphenyl ether tetracarboxylic acid dianhydride (>98%, M_p_ = 226 °C), 3,3′,4,4′-benzophenonetetracarboxylic dianhydride (>96%, M_p_ = 222 °C), 4,4′-(hexafluoroisopropylidene)diphthalic anhydride (98%, M_p_ = 244–245 °C), 1,4,5,8-naphthalenetetracarboxylic dianhydride (97%), pyromellitic dianhydride (98%, M_p_ = 285–287 °C) were purified by vacuum sublimation (20 Pa) at the temperatures close to their melting points. 4,4′-(4,4′-Isopropylidenediphenoxy)bisphthalic dianhydride (97%, M_p_ = 184,5–186 °C) was dried at 140 °C in vacuum for 8 h. Terephthaloyl chloride (>98%, M_p_ = 83 °C) was purified by vacuum distillation before use. 3,3-Bis(4-aminophenyl)phthalide (M_p_ = 204 °C) was recrystallized from ethanol. Pyridine (99%) and acetic anhydride (98%) were used as received without further purification. NBP (99%) and NMP (99%) were kept for two days over CaH_2_ and then distilled under reduced pressure before use. According to Fischer’s analysis, the moisture content in NBP and NMP was 0.014% and 0.020%, respectively. All other commercially available reagent-grade chemicals were used without additional purification.

### 3.2. Polyimide Synthesis

PIs were synthesized by one-step or two-step methods. Synthesis of ODPA-AFL is given below as an example. The structures of the synthesized PIs have been confirmed by FT-IR spectroscopy (Supporting Information, Figures S1–S10).

#### 3.2.1. One-Step Method

ODPA (1.5511 g, 5 mmol), AFL (1.7423 g, 5 mmol) and 12.5 mL NBP were charged into a 100 mL, a tree-necked round-bottomed flask. The mixture was heated to 180 °C in an inert atmosphere with stirring. The reaction was continued for 5 h at this temperature. Aliquots were taken from the reaction mixture at specific time intervals (0.5, 1, 2, 3 and 5 h) and a part of the polymer was precipitated into an excess of methanol to estimate the change in the molecular weight. The samples were washed in the Soxhlet extractor with methanol for 2 days and then dried under normal conditions for 24 h, in a vacuum at 70 °C for two days, and finally at 220 °C during 1 h.

#### 3.2.2. Two-Step Methods

ODPA-AFL polyimide was synthesized via the two-step methods through a PAA intermediate.

##### Synthesis of PAA

AFL (3.4845 g, 10.0 mmol) was dissolved in 27.5 mL of NBP at room temperature. The solution was then cooled to 10 °C in an ice bath. Under an inert argon atmosphere, a stoichiometric amount of ODPA (3.1022 g, 10.0 mmol) was added slowly to the cooled diamine solution. The reaction mixture was stirred at 10 °C for 7 h, followed by 8 h at ambient temperature (25 °C), yielding a 20 wt% PAA solution in NBP. This PAA precursor was divided into three equal parts for subsequent imidization via different methods.

##### Chemical Imidization

A portion of the PAA solution was chemically imidized by adding pyridine (catalyst, 1.75 molar excess) and acetic anhydride (dehydrating agent, twofold molar excess). The mixture was stirred under an inert atmosphere at 25 °C for 2.5 h and then at 80 °C for 2 h. The resulting viscous polyimide solution was diluted with NMP to approximately 5 wt% and precipitated into a fivefold excess of vigorously stirred deionized water. The fibrous precipitate was collected by filtration, washed with deionized water several times, and then in a Soxhlet extractor with methanol for 48 h. Polyimide was dried under normal conditions for 24 h, in a vacuum at 70 °C for two days, and finally at 220 °C during 1 h.

##### Thermal Imidization

A portion of the 20 wt% PAA solution was cast onto a clean glass plate. PAA was then thermally treated at 100 °C, 150 °C, and 200 °C for 1 h at each temperature. Subsequently, the film was detached from the substrate and annealed in a furnace at 200 °C, 300 °C, and 400 °C (1 h each) to complete the imidization cyclization and remove any residual solvent.

##### Thermal Imidization with an Azeotropic Mixture

A portion of the PAA solution was thermally imidized in solution using *o*-dichlorobenzene as a co-solvent to form an azeotrope with the water released during imidization. *o*-Dichlorobenzene (30 vol% relative to NBP) was added to the PAA solution. The mixture was heated at 210 °C under argon with vigorous stirring for 16 h. The resulting polyimide solution was diluted with NMP to about 5 wt% and precipitated into a fivefold excess of methanol. The polymer was collected by filtration, washed in a Soxhlet extractor with methanol for 48 h, and dried under normal conditions for 24 h, in a vacuum at 70 °C for two days, followed by 220 °C for 1 h.

The structures of all PIs obtained have been confirmed by FT-IR spectroscopy (Supporting Information, Figures S1–S10).

### 3.3. Polyamide Synthesis

PAs were obtained by low-temperature polycondensation of the diamine with terephthaloyl chloride. Synthesis of FDA:TPC is given below as an example. In a 250 mL three-neck flask, equipped with a mechanical stirrer and argon in-let, 3.10 g (10 mmol) of FDA was dissolved in 20 mL of NBP at room temperature. The flask was cooled to −30 °C using a liquid nitrogen/acetone bath, and then solid terephthaloyl chloride (2.03 g, 10 mmol) was added to the reaction mixture in portions. The reaction was conducted at −30 °C for 3 h and then stirred at room temperature for 1 h. The resulting highly viscous PA solution was diluted with NMP up to 5 wt%. Polymer was precipitated into water. Fiber-like precipitate was collected by filtration, washed in the Soxhlet extractor with acetone for 2 days and then dried in a vacuum at 70 °C (6 h), 150 °C (6 h) and finally at 250 °C (1 h). Yield: 97%, 4.25 g. The structures of the synthesized PAs have been confirmed by FT-IR spectroscopy (Supporting Information, Figures S11 and S12)

### 3.4. Poly(Amide-Imide) Synthesis

PAIs were synthesized in the same manner, according to the method described in detail earlier [5,71]. The synthesis of ODPA:FDA:TPC illustrates the general synthetic route. In a 250 mL three-neck flask, equipped with a mechanical stirrer and argon in-let, 3.10 g (10 mmol) of FDA was dissolved in 20 mL of NBP at room temperature. ODPA (1.55 g, 5.0 mmol) was added to the solution cooled up to 10 °C and the reaction mixture was stirred at this temperature until the formation of a homogeneous solution and for further 1–5 h. Then the reaction mixture was cooled to −30 °C using a liquid nitrogen/acetone bath, and 1.02 g (5.0 mol) of solid TPC was added portionwise. The reaction was carried out for another 2 h. The chemical imidization of polyamide-amic acid was performed using a mixture of acetic anhydride (3.7 mL, 40 mmol) and pyridine (2.8 mL, 35 mmol) at room temperature during 12 h and finally at 60 °C for 4 h. The resulting highly viscous PAI solution was diluted with NMP up to 5 wt%. Polymer was precipitated into 1.5 L of water under continuous stirring. Fiber-like precipitate was collected by filtration, washed thoroughly several times with water and methanol, then dried in vacuum oven at 70 °C (6 h), 150 °C (6 h) and finally at 250 °C (1 h). Yield: 96%, 4.92 g. The structures of PAIs have been confirmed by FT-IR spectroscopy (Supporting Information, Figures S13–S16).

### 3.5. Methods

FT-IR spectra were recorded on a Perkin Elmer spectrometer (PerkinElmer Instruments Co., Ltd., Shelton, CT, USA) using a UATR accessory Perkin Elmer (USA) with a Di/ZnSe crystal. 

The inherent viscosity (η_inh_) was measured for the solution of 0.05 g of polymer in NMP (10.0 mL) at 25.0 °C using an Ostwald viscometer. For some PAAAs, the inherent viscosities were estimated as a function of the reaction time. For this purpose, the samples taken at certain time intervals during the synthesis were diluted with dry solvent to C = 0.5 g/dL, and then the solution flow time was determined. High temperature gel permeation chromatography (GPC) analyses of polymers were performed with a Waters Alliance GPCV 2000 GPC instrument equipped with a Waters DRI detector (Waters Corporation, Milford, MA, USA). The column set (Styragel HT6E) was eluted with NMP polymer solutions (C = 1.0 mg/mL) at 1.0 mL/min at 80 °C. Data were calibrated using monomodal polystyrene standards (from Polymer Standards Service).

Thermomechanical analysis (TMA) was performed with a thermomechanical analyzer TMA/SDTA 2+ LN/600 (Mettler-Toledo, Greifensee, Switzerland) at a heating rate of 5 °C/min with constant load 1 N using a quartz stem with a ball point tip (diameter 3 mm). Thermogravimetric analysis (TGA) was performed by Thermoanalyzer Shimadzu DTG-60H (Shimadzu Corporation, Kyoto, Japan) on samples with weight of about 5 mg at a heating rate of 10 °C/min in air. The temperature at which a weight loss of 10% was detected was considered to be the decomposition onset temperature.

## 4. Conclusions

The synthesis of PIs, PAs, and PAIs bearing side cardo, trifluoromethyl, and carboxyl groups in NBP has been thoroughly investigated. It has been demonstrated that NMP can be successfully replaced by NBP in both one- and two-step syntheses of PIs. Notably, the one-step process in NBP leads to a significant acceleration in the formation of high-molecular-weight products, achieving target molecular weights within 1–2 h. This effect is presumably due to the greater hydrophobicity of NBP, which facilitates more efficient removal of water (the condensation by-product) from the reaction mixture, thereby shifting the equilibrium toward product formation.

While NBP proved to be highly effective for synthesizing various PIs with five-membered imide cycles, its application for polymers with six-membered cycles based on 1,4,5,8-naphthalenetetracarboxylic dianhydride was limited. In the latter case, precipitation occurred during the reaction, resulting in low-molecular-weight products.

For PAs and PAIs, the choice of NBP generally yielded polymers with slightly lower molecular weights and required longer reaction times compared to the reference polymers produced in NMP. This difference in kinetics and achievable molecular weight is influenced by the solvent’s physicochemical properties, such as its Kamlet–Taft parameters and dielectric constant, which affect both the main reaction rate and the likelihood of side reactions. Optimization revealed that a 7 mol.% excess of terephthaloyl chloride significantly increases the molecular weights of PAIs obtained in NBP.

Furthermore, the choice of solvent impacts the physical form and solubility of the products. Using NBP for the synthesis of fluorinated PAs or PAIs resulted in the formation of hard yellowish pellets soluble only in hot organic solvents. In contrast, the same polymers isolated from NMP were obtained as white fibers with high solubility at room temperature. The glass transition temperatures and thermo-oxidative stability of all polymers were found to be independent of the solvent applied.

In summary, NBP—a less toxic and more environmentally benign alternative to traditional solvents such as NMP, DMAA, DMF, and *m*-cresol—has been established as a promising reaction medium for obtaining high-molecular-weight PIs, PAs, and PAIs. We propose that NBP is suitable not only for the classes of polymers studied here but also holds potential for the production of other high-performance polymers, including polysulfones, polyetherketones, polybenzimidazoles, and polyarylates.

## Figures and Tables

**Table 1:** Properties of PIs obtained by one-step synthesis in NBP.

PI	Time, h	η_inh_, dL/g	M_n_, kDa	M_w_, kDa	M_w/_M_n_	T_g,_ °C	T_10%_, °C
ODPA-AFL	5	0.7	56	169	3.0	350	550
ODPA-AFL ^1^	20	0.8	62	164	2.7	360	550
ODPA-AFL ^2^	31	0.7	37	133	3.6	350	540
ODPA-AFL ^3^	21	-	-	-	-	-	550
ODPA-AFL ^4^	7	0.4	17	69	4.2	350	510
ODPA-AFL_0.5_:DABA_0.5_	4	1.1	254	462	1.8	335	530
ODPA-AFL_0.5_:DABA_0.5_^4^	6	1.0	227	449	2.0	340	540
BTDA-AFL_0.5_:DABA_0.5_	7	0.9	91	398	4.4	340	520
BTDA-AFL_0.5_:DABA_0.5_^4^	7	1.0	146	421	2.9	340	510
6FDA-DABA	6	0.7	275	430	1.6	330	500
6FDA-DABA ^4^	6	0.8	346	537	1.6	335	490
BPADA-DABA	6	0.8	239	440	1.8	260	470
PMDA-APH	4	0.9	191	320	1.7	-	510
NTDA-APH	5	0.3	12	26	2.2	-	510
NTDA-APH_0.5_:DABA_0.5_	4	0.2	12	56	4.8	-	495

^1^ Two-step synthesis via chemical imidization. ^2^ Two-step synthesis by thermal imidization with an azeotropic agent. ^3^ Two-step synthesis by thermal imidization. ^4^ One-step synthesis in NMP.

**Table 2:** Properties of PAs obtained in NBP or NMP.

Polymer	Solvent	η_inh_, dL/g	M_n_, kDa	M_w_, kDa	M_w_/M_n_	Solubility ^1^		T_g_, °C	T_10%_ °C
						S1	S2		
TPC-AFL	NBP	0.9	46	137	2.9	+	−	380	470
	NBP ^2^	0.7	-	-	-	-	-	-	-
	NBP ^3^	0.4	-	-	-	-	-	-	-
	NMP	1.8	165	354	2.1	+	−	380	500
TPC-TFMB	NBP	2.3	51	164	3.2	±	−	260	500
	NMP	3.0	44	199	4.5	+	−	260	480

^1^ S1: NMP, NBP, DMAA, DMF; S2: DMSO. “+”—Soluble at room temperature; “±”—soluble upon heating; “−”—insoluble. ^2,3^ 3 and 7 mol.% excess of TPC was taken.

**Table 3:** The inherent viscosities of PAAAs and final PAIs.

Polymer	Solvent	η_inh_, dL/g	
		PAAA	PAI
ODPA:TPC-AFL	NBP	0.7	0.6
	NMP	1.2	0.9
BTDA:TPC-AFL	NBP	0.4	0.3
	NMP	0.7	0.6
ODPA:TPC-TFMB	NBP	1.6	1.6
	NMP	2.6	2.6
BTDA:TPC-TFMB	NBP	1.4	1.3
	NMP	2.0	2.0

**Table 4:** The effect of TPC excess on the inherent viscosities of PAAAs and final PAIs.

Polymer	Solvent	η_inh_ of PAAA/PAI (Excess of TPC, mol.%), dL/g			
		0	3	7	10
ODPA:TPC-AFL	NBP	0.7/0.6	0.8/0.7	0.9/0.9	0.9/0.8
	NMP	1.2/0.9	1.1/0.9	1.1/0.9	-
BTDA:TPC-AFL	NBP	0.4/0.3	0.6/0.5	0.8/0.7	0.8/0.7
	NMP	0.7/0.6	0.7/0.6	0.8/0.6	-

**Table 5:** Properties of PAIs obtained in NBP or NMP.

Polymer	Solvent	η_inh_, dL/g	M_n_, kDa	M_w_, kDa	M_w_/M_n_	Solubility ^1^		T_g_, °C	T_10%_, °C
						S1	S2		
ODPA:TPC-AFL	NBP ^2^	0.9	57	213	3.7	+	±	370	515
	NMP	0.9	91	250	2.7	+	±	370 ^3^	500 ^4^
BTDA:TPC-AFL	NBP ^2^	0.7	46	195	4.2	+	±	380	500
	NMP	0.6	30	129	4.2	+	±	380 ^3^	490 ^4^
ODPA:TPC-TFMB	NBP	1.6	119	316	2.7	+	+	270	500
	NMP	2.6	211	472	3.0	+	+	260 ^3^	510 ^4^
BTDA:TPC-TFMB	NBP	1.3	202	384	1.9	±	±	285	525
	NMP	2.0	166	412	2.4	+	+	280 ^3^	510 ^4^

^1^ S1: NMP, NBP, DMAA. S2: DMF, DMSO. “+”—Soluble at room temperature; “±”—soluble upon heating. ^2^ 7 mol.% excess of TPC was taken. ^3,4^ T_g_ and T_10%_ data were taken from [5].

## Data Availability

The original contributions presented in this study are included in the article/Supplementary Materials. Further inquiries can be directed to the corresponding author.

## Supplementary Materials

The following supporting information can be downloaded at: https://www.mdpi.com/article/10.3390/ijms27031252/s1.
