# Supplementary material for: Synthesis of Polyimides, Polyamides, and Poly(Amide-Imides) in the “Green” Solvent N-Butyl-2-Pyrrolidone (TamiSolve NxG): Features, Optimization, and Versatility
Source: Int J Mol Sci. 2026 Jan 27;27(3):1252. doi: 10.3390/ijms27031252 (PMC12898531; doi:10.3390/ijms27031252)
Supplement: Supplementary file 1 [file ijms-27-01252-s001.zip › ijms-4096586-supplementary.pdf]

Supporting Information

for

Synthesis of Polyimides, Polyamides, and Poly(amide-imides) in the “Green” Solvent N-butyl-2-pyrrolidone (TamiSolve NxG): Features, Optimization, and Versatility

Olesya N. Zabegaeva, Alexander V. Chuchalov, Dmitriy A. Khanin, Denis O. Ponkratov and Dmitriy A. Sapozhnikov*

A.N. Nesmeyanov Institute of Organoelement Compounds, Russian Academy of Sciences, Vavilov str. 28, Moscow 119334, Russia.

* Corresponding author: ssddaa@ineos.ac.ru

Table of Contents

FT-IR spectra S2

GPC results S18

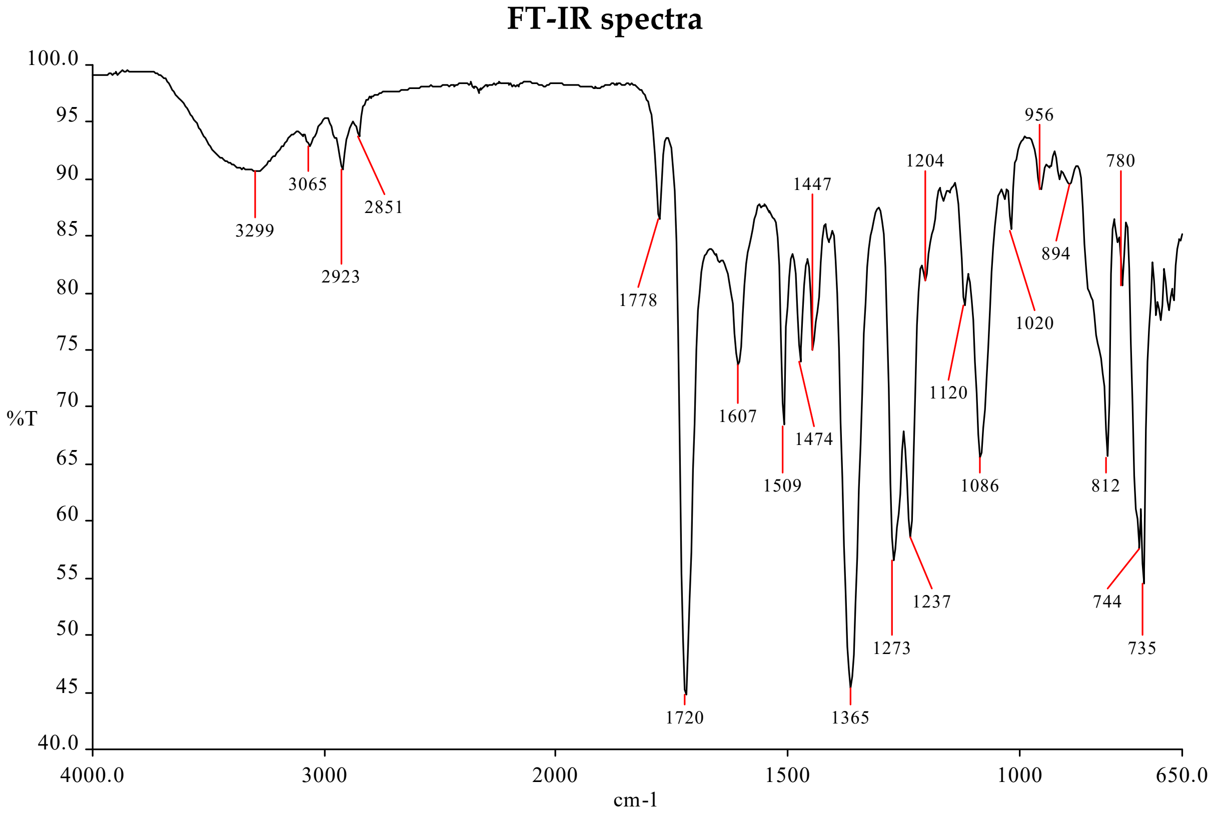

ν, cm⁻¹	Bonds	ν, cm⁻¹	Bonds
1778	ν_{as}(C=O) imide	1272	ν(C-O-C)
1720	ν_{s}(C=O) imide	1235	
1607	ν(C=C) ring	1120	ν(C-C)
1509	ν(C=C) fluorene	1086	ν(CNC) imide
1474	δ(C-O)	812	ω(CH) in CH=CH
1447	δ(CH)	744	δ(CO)
1365	ν(CNC) imide	735	ν(CNC) imide

Figure S1. FT-IR spectrum of ODPA-AFL (one-step, NBP).

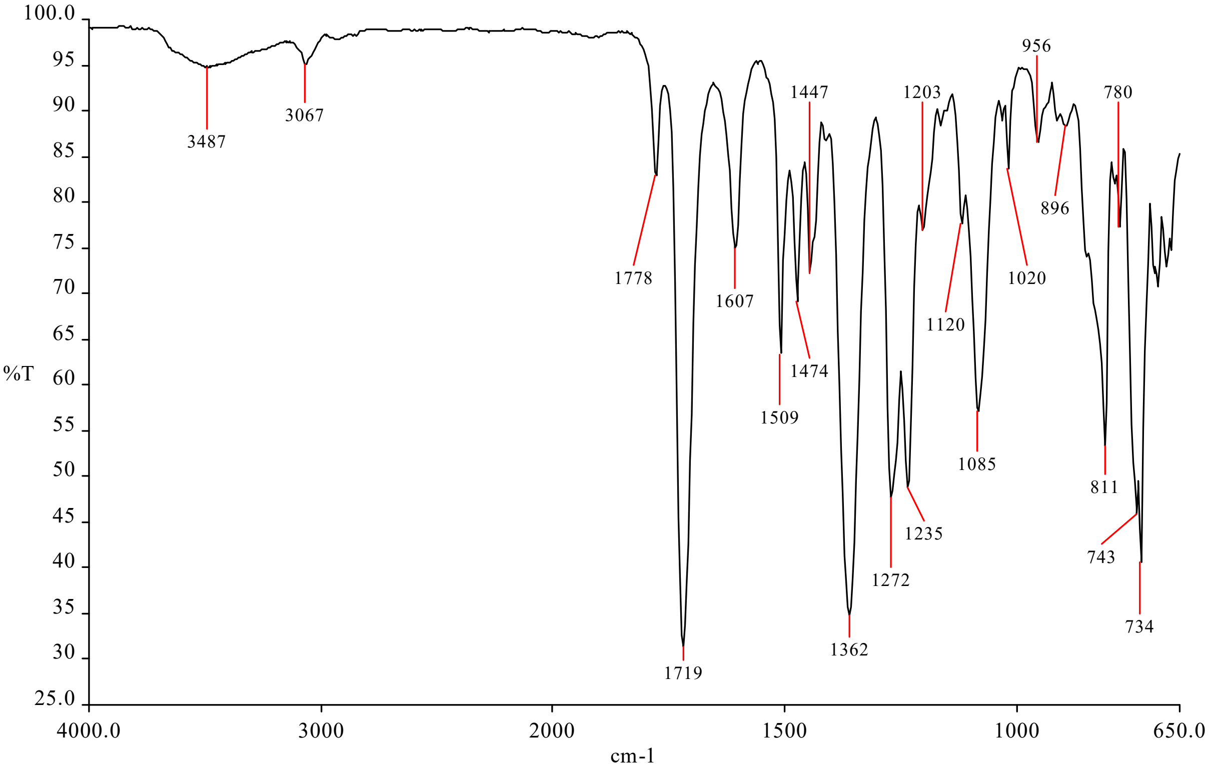

ν, cm^{-1}	Bonds	ν, cm^{-1}	Bonds
1778	ν_{as}(C=O) imide	1272	ν(C-O-C)
1720	ν_{s}(C=O) imide	1235	
1607	ν(C=C) ring	1120	ν(C-C)
1509	ν(C=C) fluorene	1085	ν(CNC) imide
1474	δ(C-O)	812	ω(CH) in CH=CH
1447	δ(CH)	744	δ(CO)
1365	ν(CNC) imide	735	ν(CNC) imide

Figure S2. FT-IR spectrum of ODPA-AFL (two-step, chemical imidization).

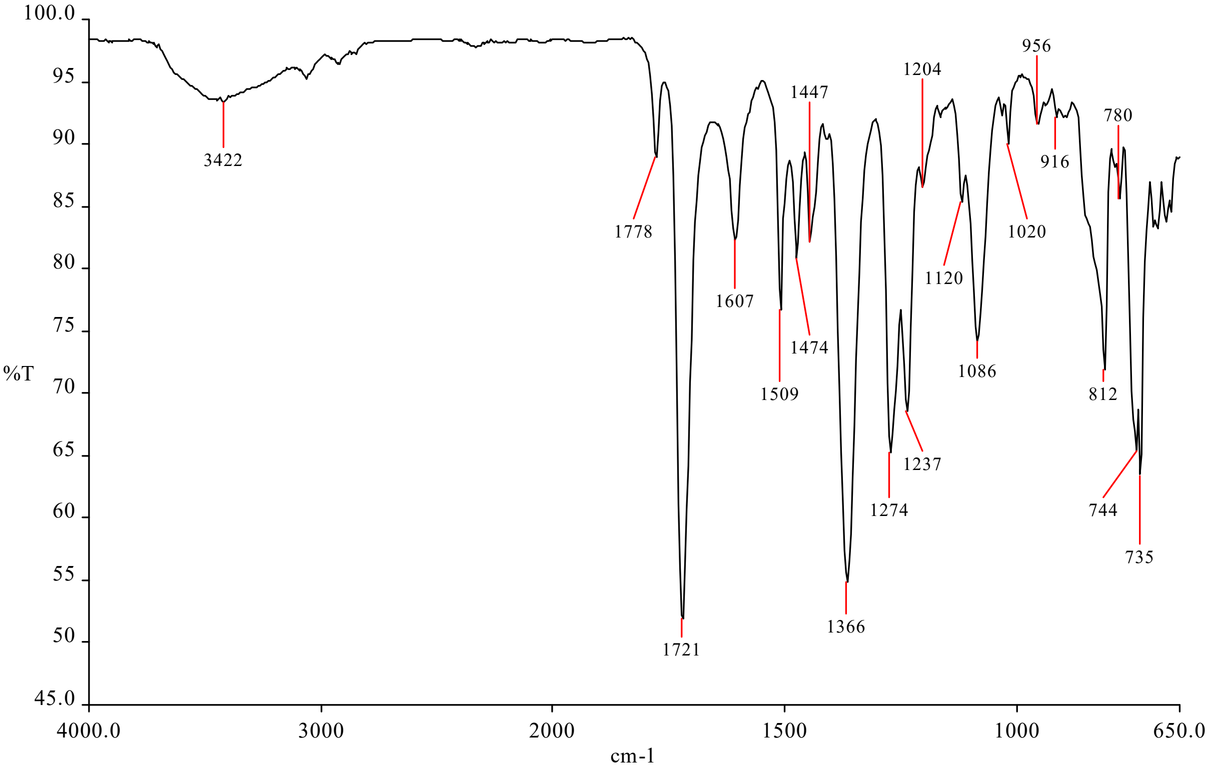

ν, cm⁻¹	Bonds	ν, cm⁻¹	Bonds
1778	ν_{as}(C=O) imide	1274	ν(C-O-C)
1720	ν_{s}(C=O) imide	1237	
1607	ν(C=C) ring	1120	ν(C-C)
1509	ν(C=C) fluorene	1086	ν(CNC) imide
1474	δ(C-O)	812	ω(CH) in CH=CH
1447	δ(CH)	744	δ(CO)
1365	ν(CNC) imide	735	ν(CNC) imide

Figure S3. FT-IR spectrum of ODPA-AFL (two-step, thermal imidization with an azeotropic agent).

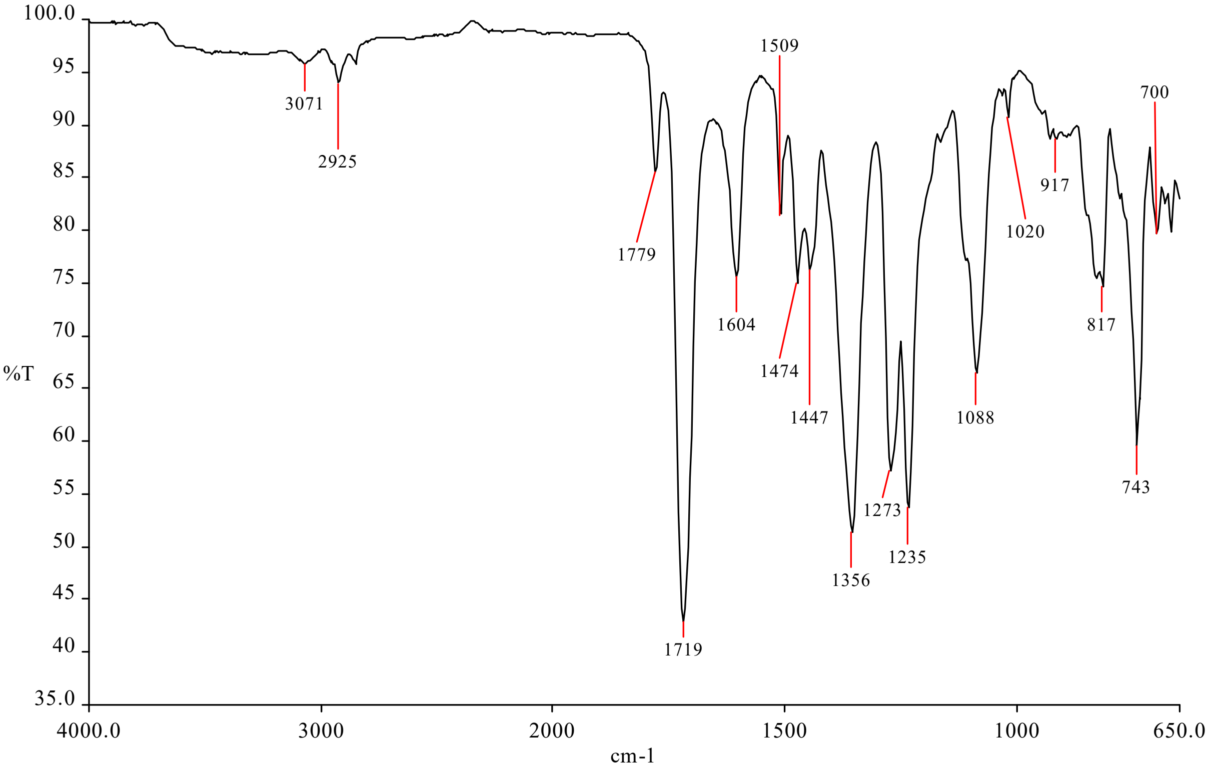

ν, cm^{-1}	Bonds	ν, cm^{-1}	Bonds
2700-3600	ν(OH)	1356	ν(CNC) imide
1779	ν_{as}(C=O) imide	1273	
1719	ν_{s}(C=O) imide, ν(C=O) acid	1235	ν(C-O-C)
1604	ν(C=C) ring	1088	ν(CNC) imide
1509	ν(C=C) fluorene	1020	γ(CH)
1474	δ(CH), δ(C-O)	817	ω(CH) in CH=CH
1447	δ(CH)	743	ν(CNC) imide

Figure S4. FT-IR spectrum of ODPA-$AFL_{0.5}$:$DABA_{0.5}$.

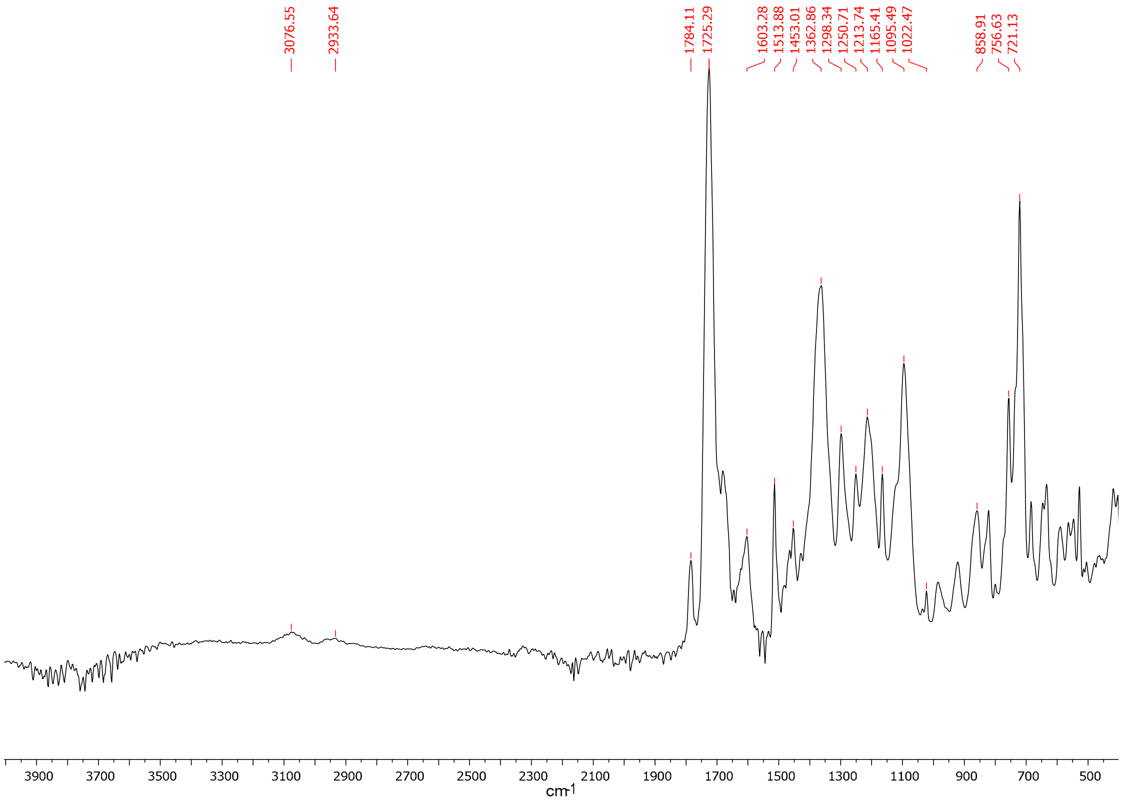

ν, cm^{-1}	Bonds	ν, cm^{-1}	Bonds
2700-3600	ν(OH)	1362	ν(CNC) imide
1784	ν_{as}(C=O) imide	1298	δ(CH)
1725	ν_{s}(C=O) imide, ν(C=O) acid	1095	ν(CNC) imide
1674	ν_{s}(C=O) BTDA	1022	γ(CH)
1513	ν(C=C) fluorene	858	ω(CH) in CH=CH
1453	δ(CH)	721	ν(CNC) imide

Figure S5. FT-IR spectrum of BTDA-$AFL_{0.5}$:$DABA_{0.5}$.

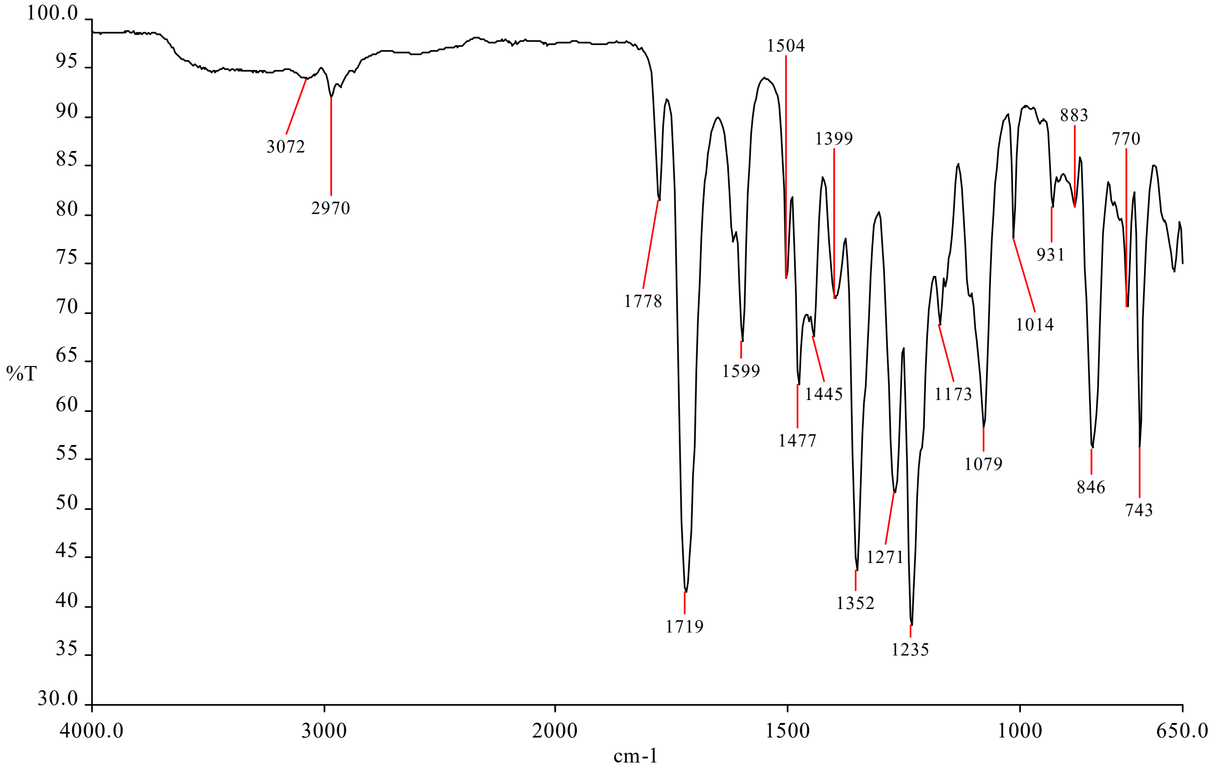

ν, cm^{-1}	Bonds	ν, cm^{-1}	Bonds
2700-3600	ν(OH)	1271	ν(C-O-C)
1778	ν_{as}(C=O) imide	1235	
1719	ν_s(C=O) imide, ν(C=O) acid	1079	ν(CNC) imide
1599	ν(C=C) ring	1014	γ(CH)
1504	ν_{ring}	931	δ(ring)
1477	δ(CH), δ(C-O)	846	ω(CH) in CH=CH
1445	δ(CH)	743	ν(CNC) imide
1352	ν(CNC) imide		

Figure S6. FT-IR spectrum of BPADA-DABA.

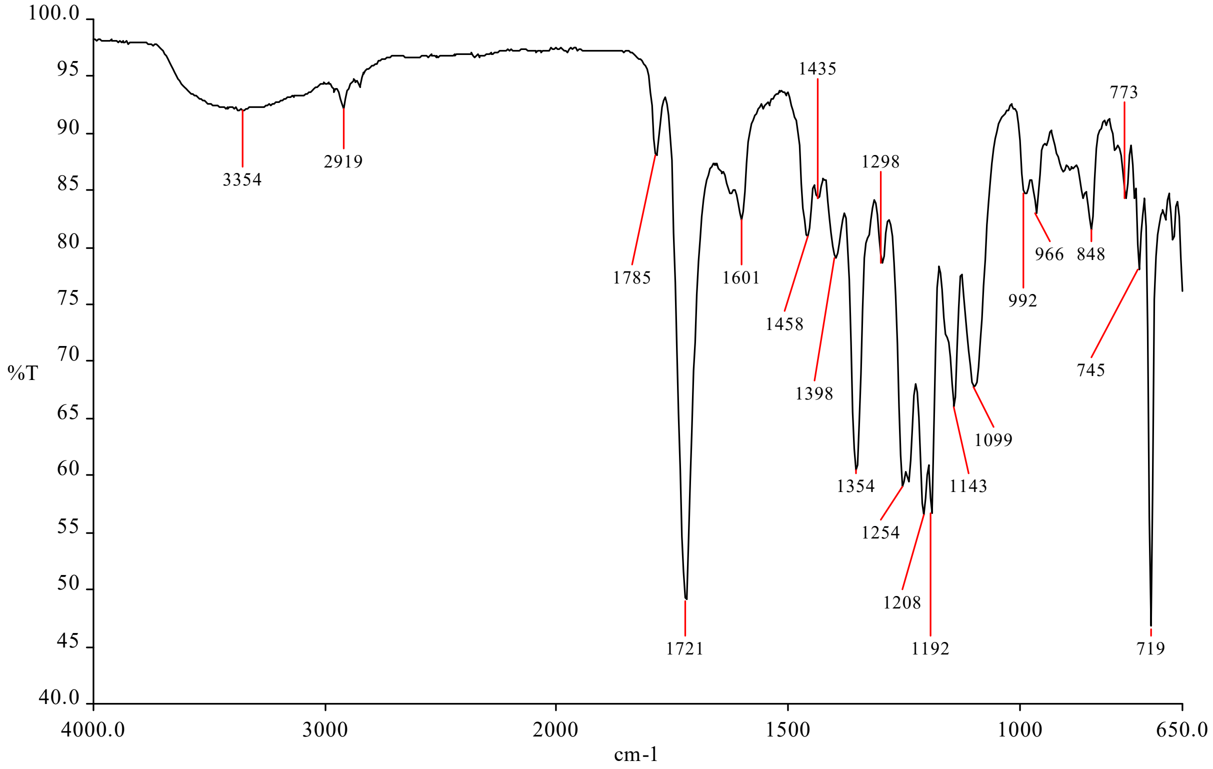

ν, cm^{-1}	Bonds	ν, cm^{-1}	Bonds
2700-3600	ν(OH)	1254	ν(C-F)
1785	ν_{as}(C=O) imide	1208	
1721	ν_{s}(C=O) imide, ν(C=O) acid	1143	ν(C-F)
1618	δ(OH)	1099	ν(CNC) imide
1601	ν(C=C) ring	992	γ(CH)
1458	δ(CH)	848	ω(CH) in CH=CH
1354	ν(CNC) imide	719	ν(CNC) imide
1298	δ(CH)		

Figure S7. FT-IR spectrum of 6FDA-DABA.

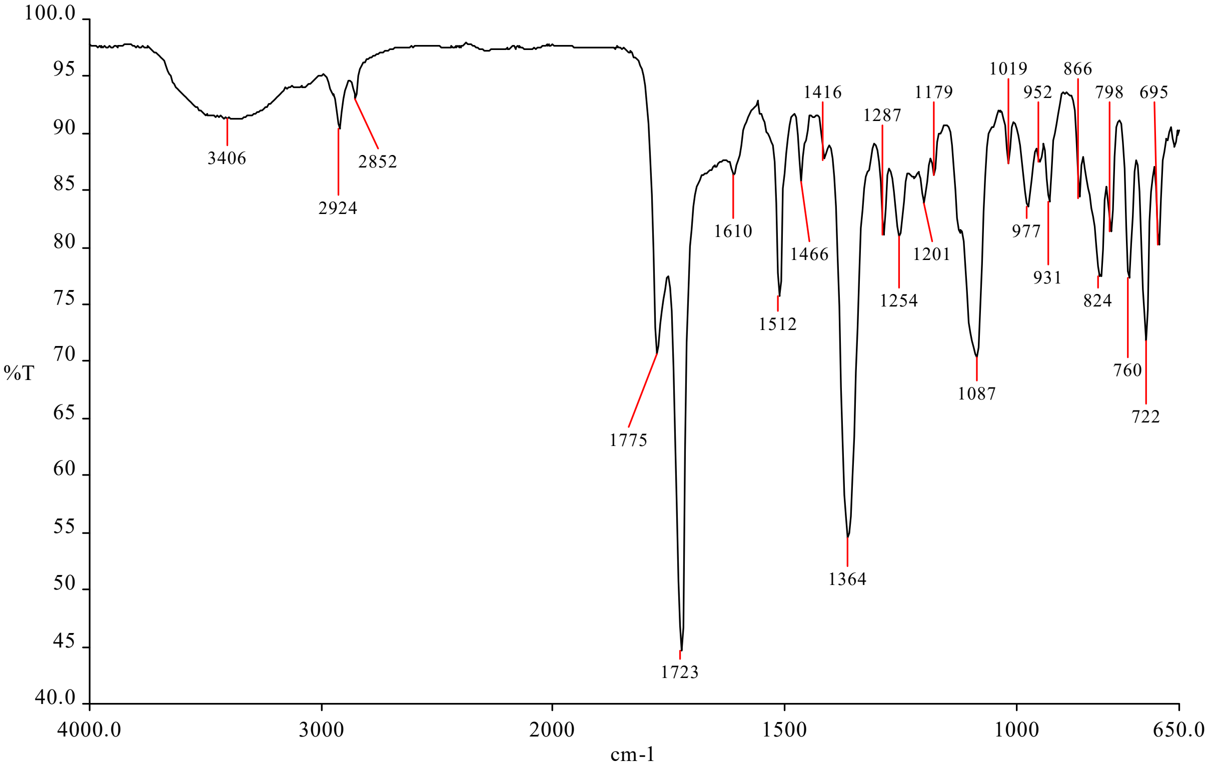

ν, cm^{-1}	Bonds	ν, cm^{-1}	Bonds
1775	ν_{as}(C=O) imide, ν(C=O) lactone	1087	ν(CNC) imide
1723	ν_{s}(C=O) imide	931	δ(ring)
1610	ν(C=C) ring	866	ω(CH) in CH=CH
1512	ν(C=C) fluorene	824	
1466	δ(CH)	722	ν(CNC) imide
1364	ν(CN) imide		
1287	δ(CH)		
1254			

Figure S8. FT-IR spectrum of PMDA-APH.

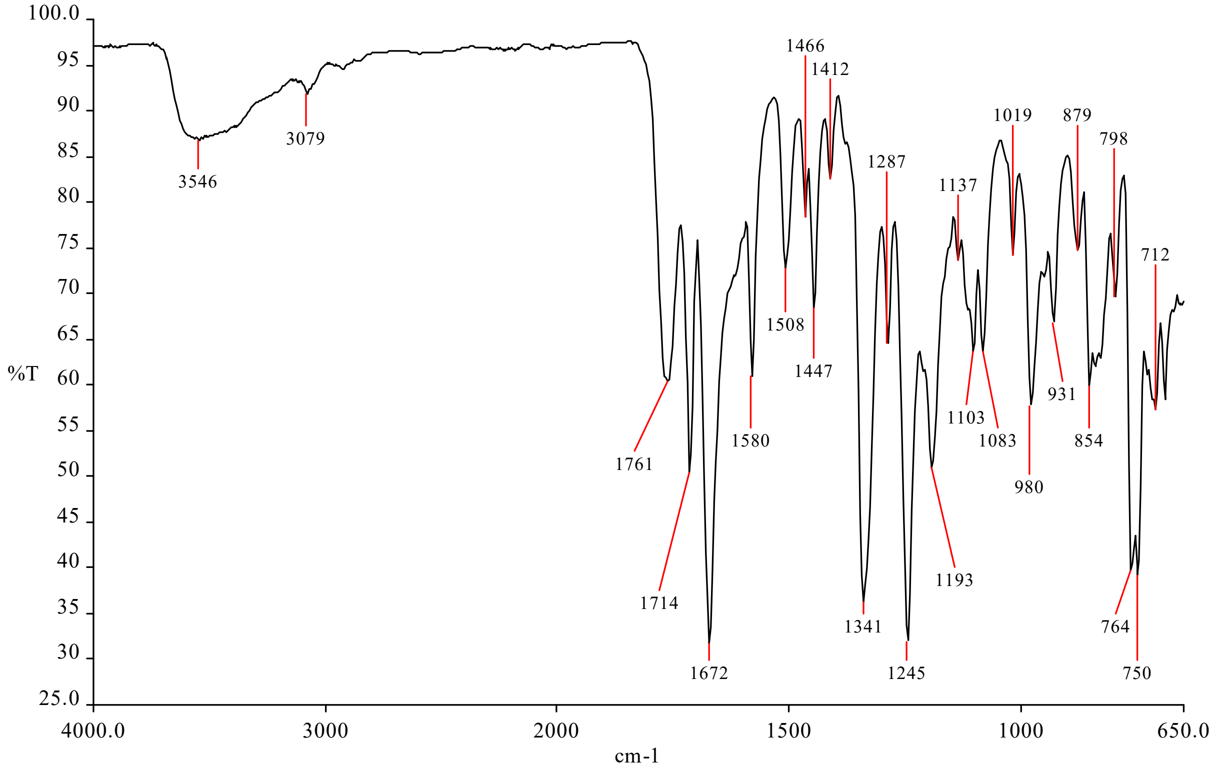

ν, cm⁻¹	Bonds	ν, cm⁻¹	Bonds
1761	ν(C=O) lactone	1103	ν(CNC) imide
1714	ν_{as}(C=O) imide	1083	
1672	ν_{s}(C=O) imide	1019	γ(CH)
1578	ν ring	931	δ(ring)
1509	ν(C=C) fluorene	854	ω(CH) in CH=CH
1447	δ(CH)	764	ν(CNC) imide
1330	ν(CNC) imide	750	

Figure S9. FT-IR spectrum of NTCDA-APH.

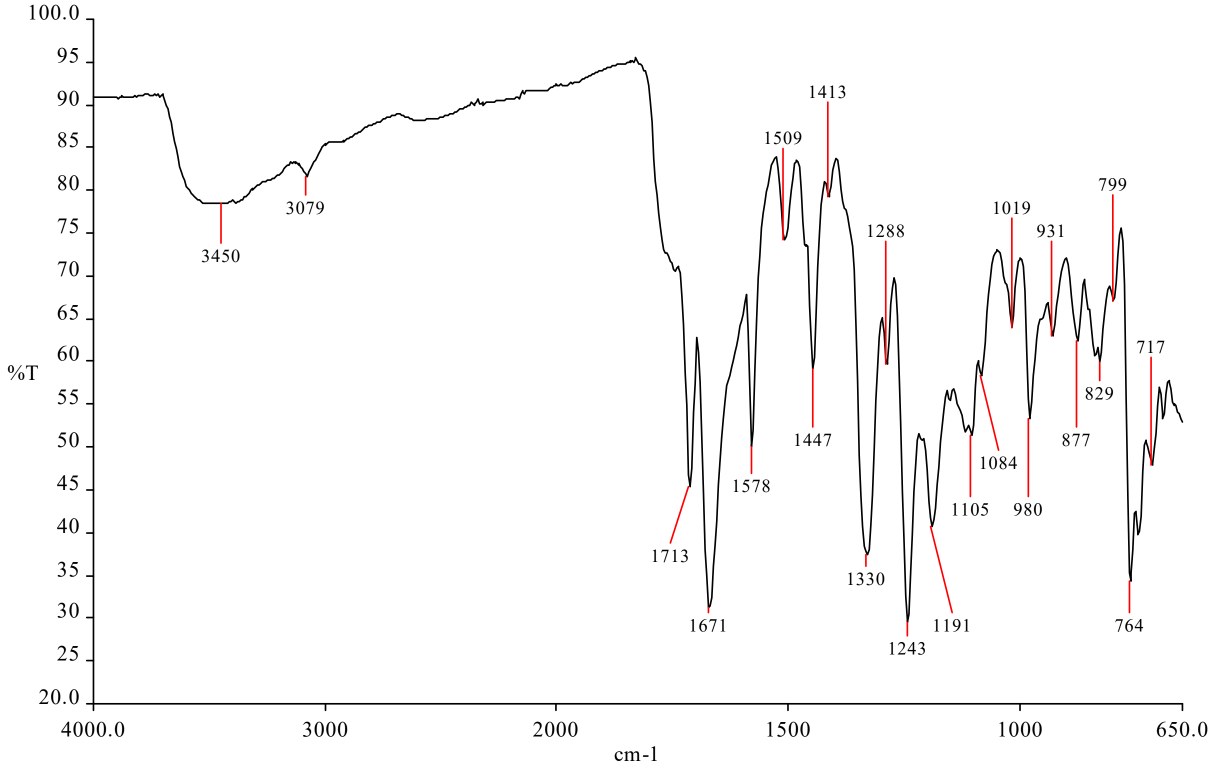

ν, cm^{-1}	Bonds	ν, cm^{-1}	Bonds
2700-3600	ν(OH)	1084	ν(CNC) imide
1760	ν(C=O) lactone	1019	γ(CH)
1713	ν_{as}(C=O) imide	931	δ(ring)
1671	ν_{s}(C=O) imide, ν(C=O) acid	854	ω(CH) in CH=CH
1509	ν(C=C) fluorene	764	ν(CNC) imide
1447	δ(CH)	750	ν(CNC) imide
1330	ν(CNC) imide	717	
1191	ν(CNC) imide		
1105			

Figure S10. FT-IR spectrum of NTCDA-$APH_{0.5}$:$DABA_{0.5}$.

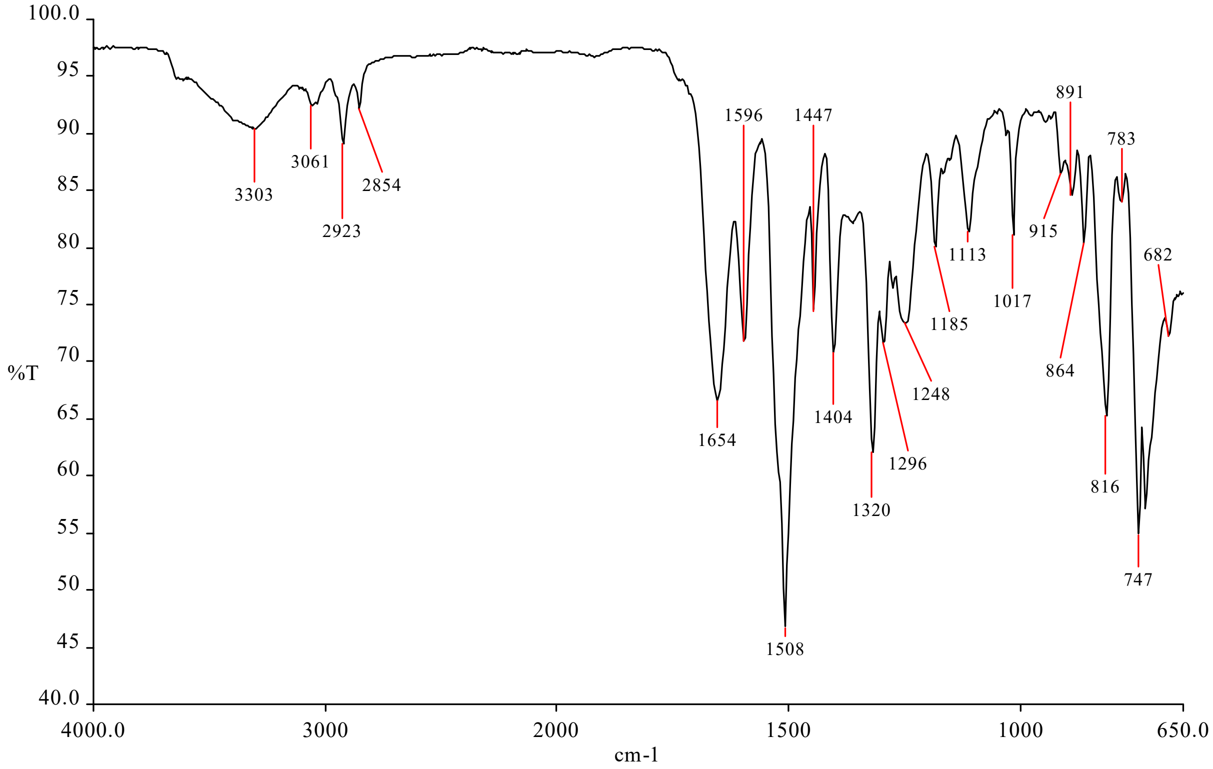

ν, cm^{-1}	Bonds
3300–3500	ν(NH)
1654	ν_s(C=O) amide
1596	ν(C=C) ring
1508	ν(C=C) fluorene

Figure S11. FT-IR spectrum of TPC-AFL.

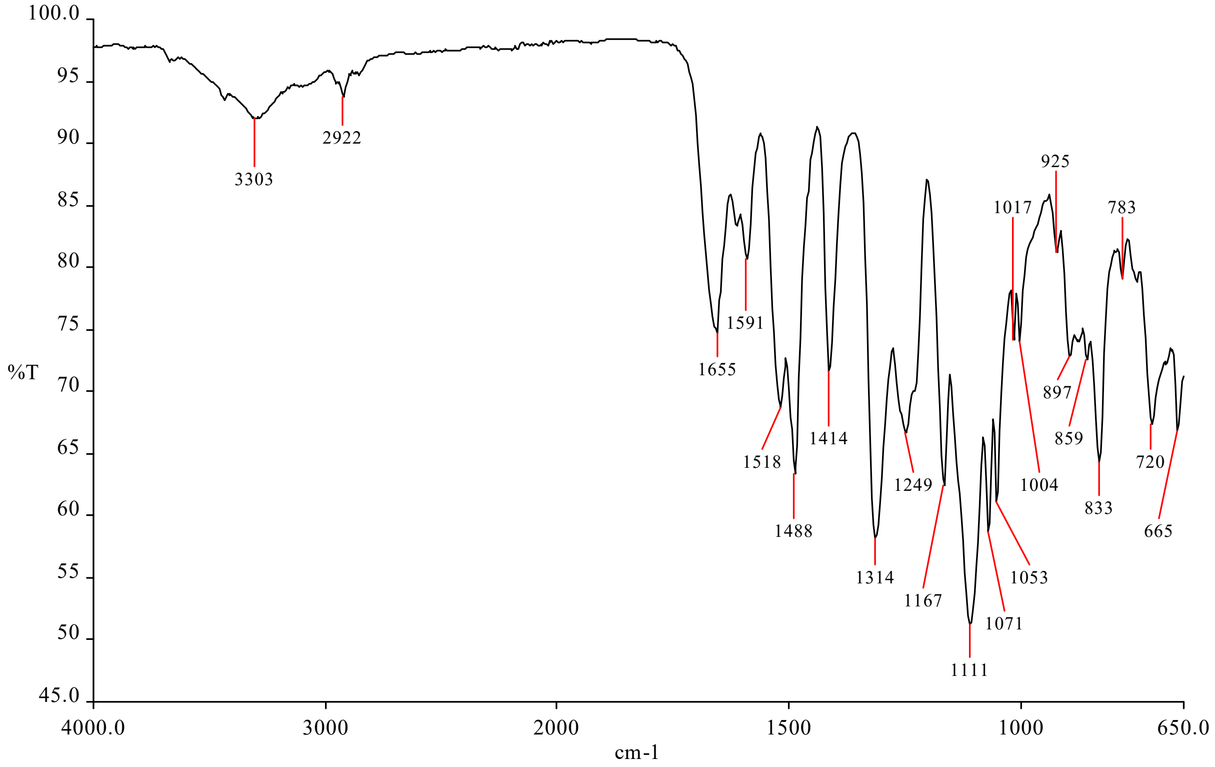

ν, cm^{-1}	Bonds
3300–3500	ν(NH)
1663	ν_s(C=O) amide
1614	δ(OH) amide
1590	ν(C=C) ring
1519	ν(CN)+δ(NH) amide

Figure S12. FT-IR spectrum of TPC-TFMB.

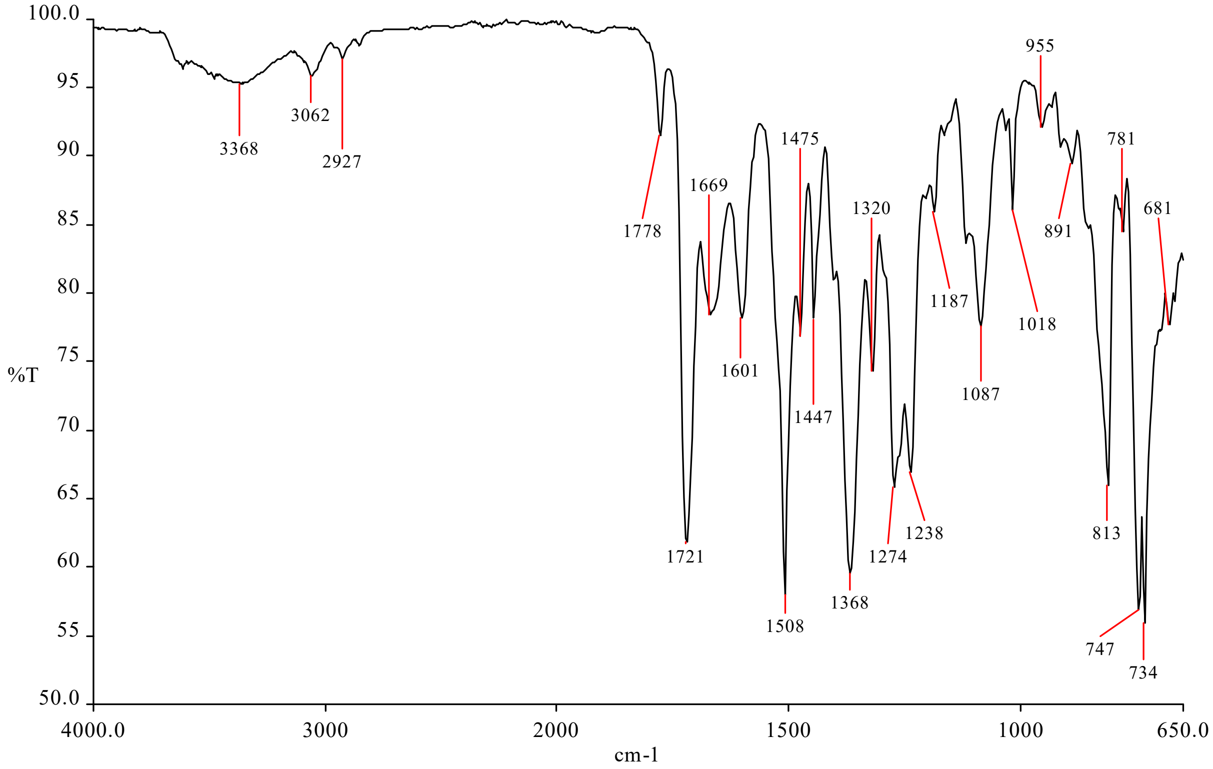

ν, cm^{-1}	Bonds
2600-3600	ν(OH)
1778	ν_{as}(C=O) imide
1721	ν_{s}(C=O) imide
1669	ν_{s}(C=O) amide
1601	ν(C=C) ring
1508	ν(C=C) fluorene
1368	ν(CNC) imide
1320	

Figure S13. FT-IR spectrum of ODPA:TPC-AFL.

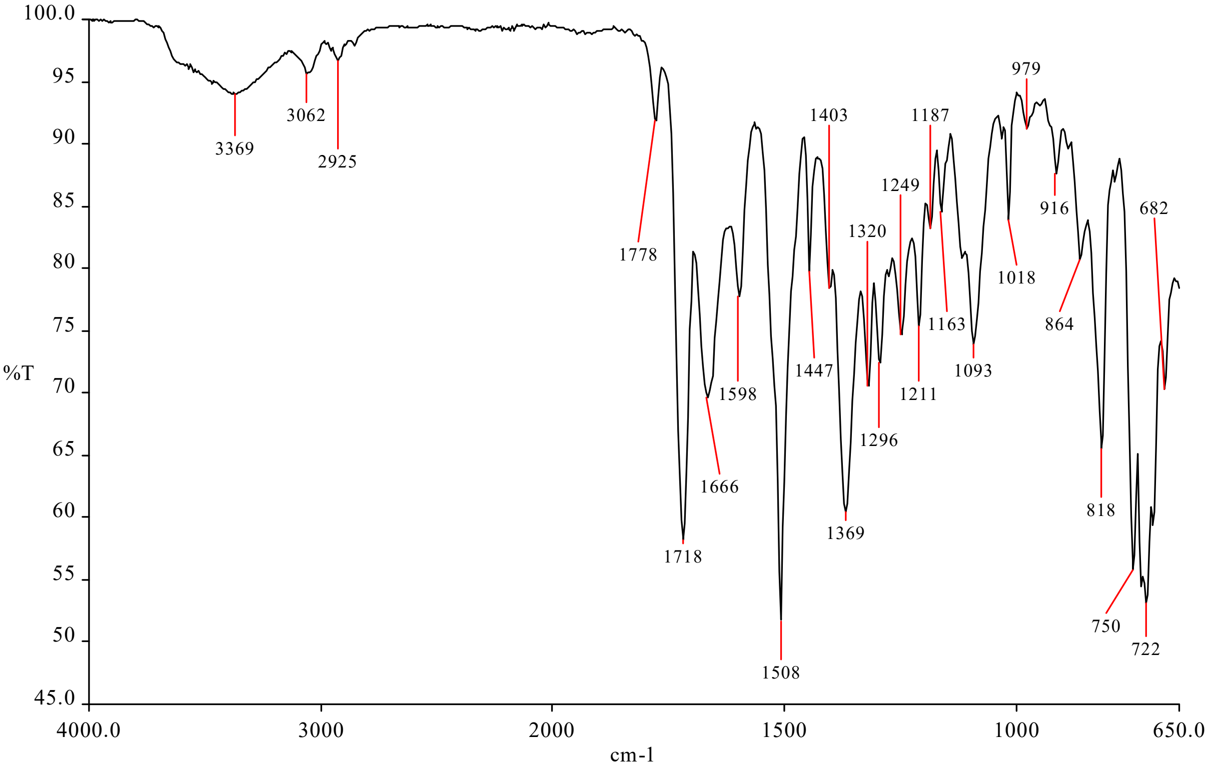

ν, cm^{-1}	Bonds
2600-3600	ν(OH)
1778	ν_{as}(C=O) imide
1718	ν_{s}(C=O) imide
1666	ν_{s}(C=O) amide
1598	ν(C=C) ring
1508	ν(C=C) fluorene
1369	ν(CNC) imide
1320	

Figure S14. FT-IR spectrum of BTDA:TPC-AFL.

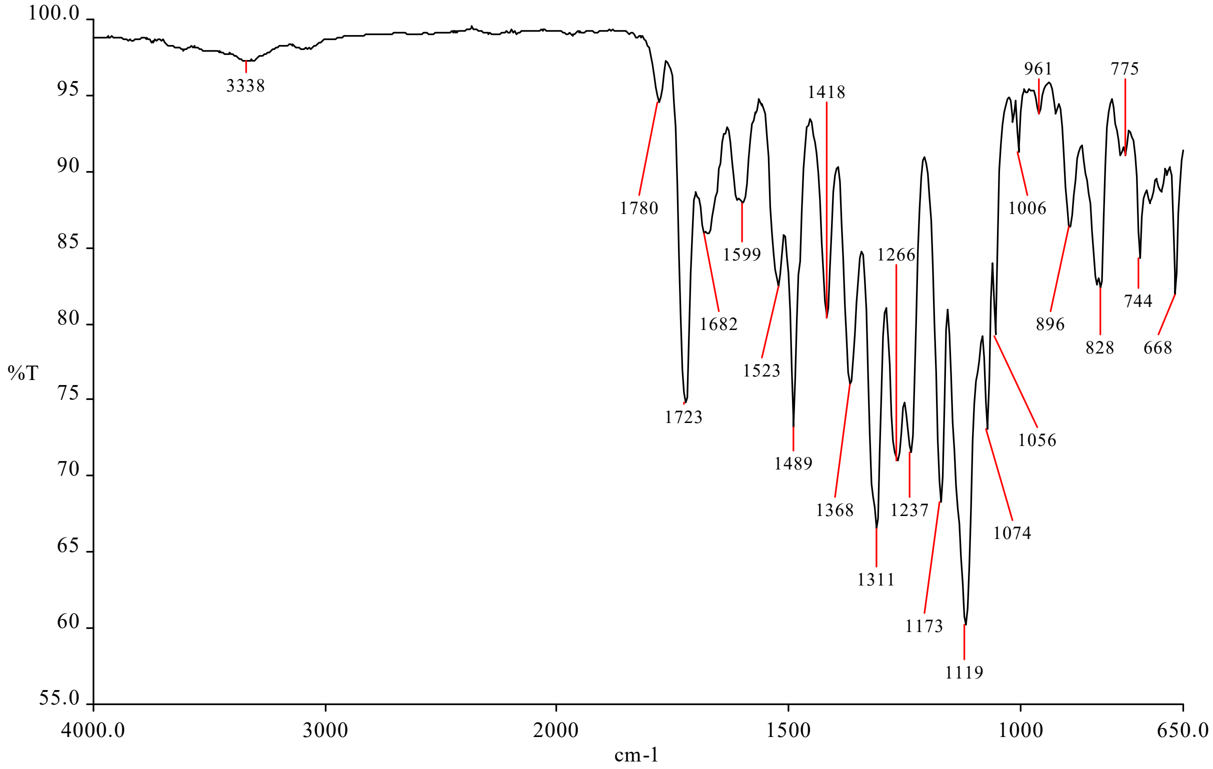

ν, cm^{-1}	Bonds
2600-3600	ν(OH)
1780	ν_{as}(C=O) imide
1723	ν_{s}(C=O) imide
1682	ν_{s}(C=O) amide
1599	ν(C=C) ring
1523	ν(CN)+δ(NH) amide
1311	ν(CNC) imide
1237	ν(C-F)
1173	
1119	
1074	

Figure S15. FT-IR spectrum of ODPA:TPC-TFMB.

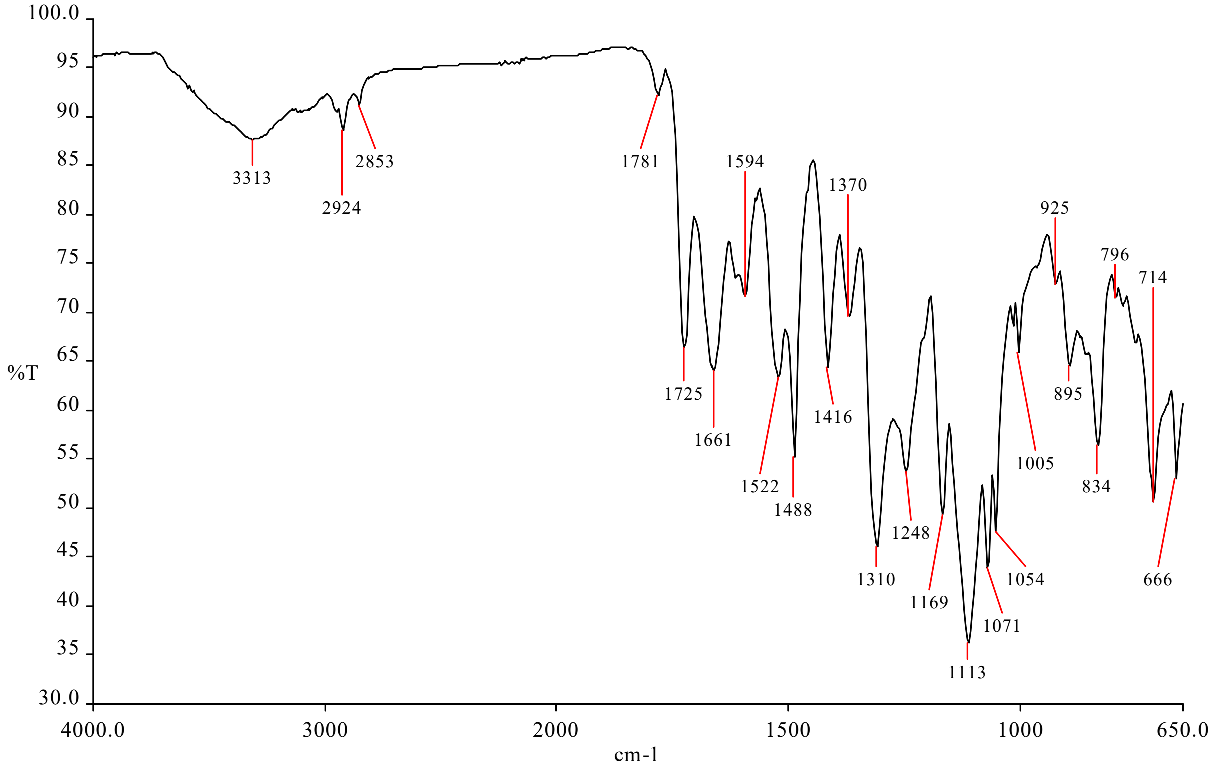

ν, cm^{-1}	Bonds
2600-3600	ν(OH)
1781	ν_{as}(C=O) imide
1725	ν_{s}(C=O) imide
1661	ν_{s}(C=O) amide
1594	ν(C=C)ring
1522	ν(CN)+δ(NH) amide
1370	ν(CNC) imide
1248	ν(C-F)
1169	
1113	
1071	

Figure S16. FT-IR spectrum of BTDA:TPC-TFMB.

GPC results

GPC Results

	Mn	Mw	MP	Mz	Mz+1	Polydispersity
1	55977	168921	184128	339230	514637	3.017689

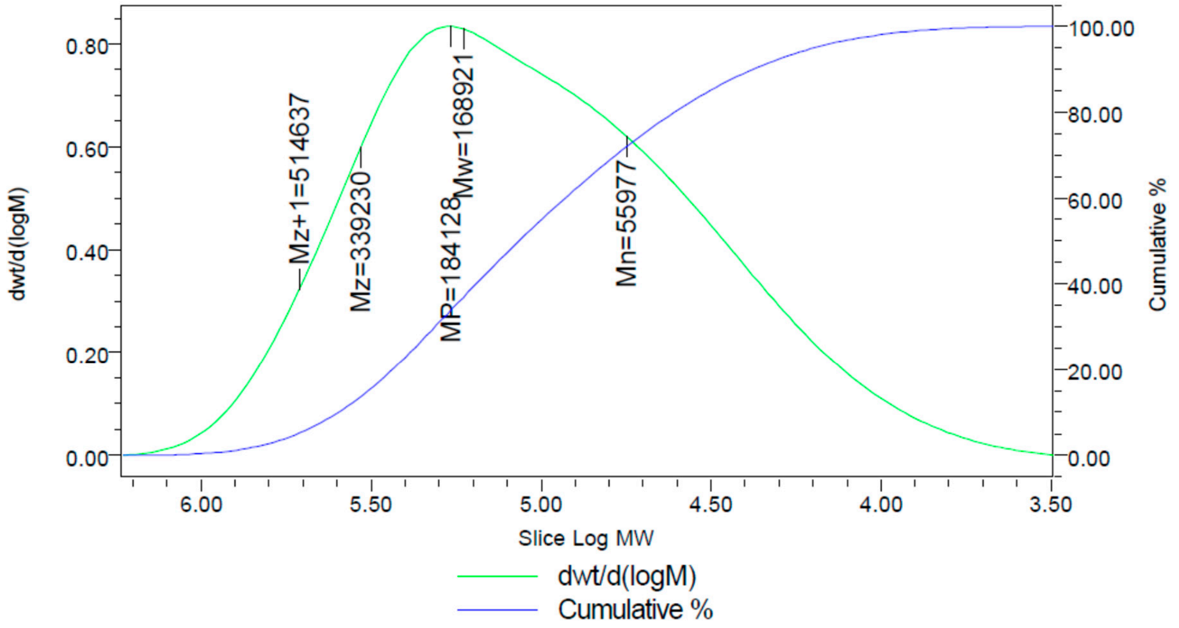

Figure S17. GPC curves and determined molecular weights of ODPA-AFL (one-step, NBP).

GPC Results

	Mn	Mw	MP	Mz	Mz+1	Polydispersity
1	61563	164435	163933	307654	445702	2.670995

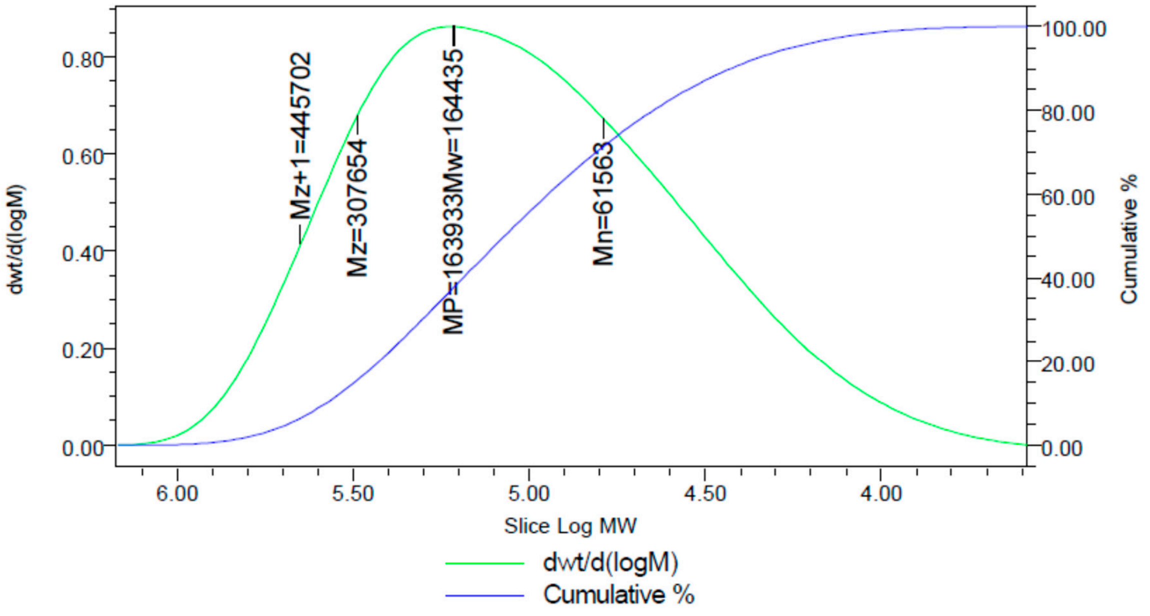

Figure S18. GPC curves and determined molecular weights of ODPA-AFL (two-step, chemical imidization).

GPC Results

	Mn	Mw	MP	Mz	Mz+1	Polydispersity
1	36788	133270	103861	274737	415724	3.622659

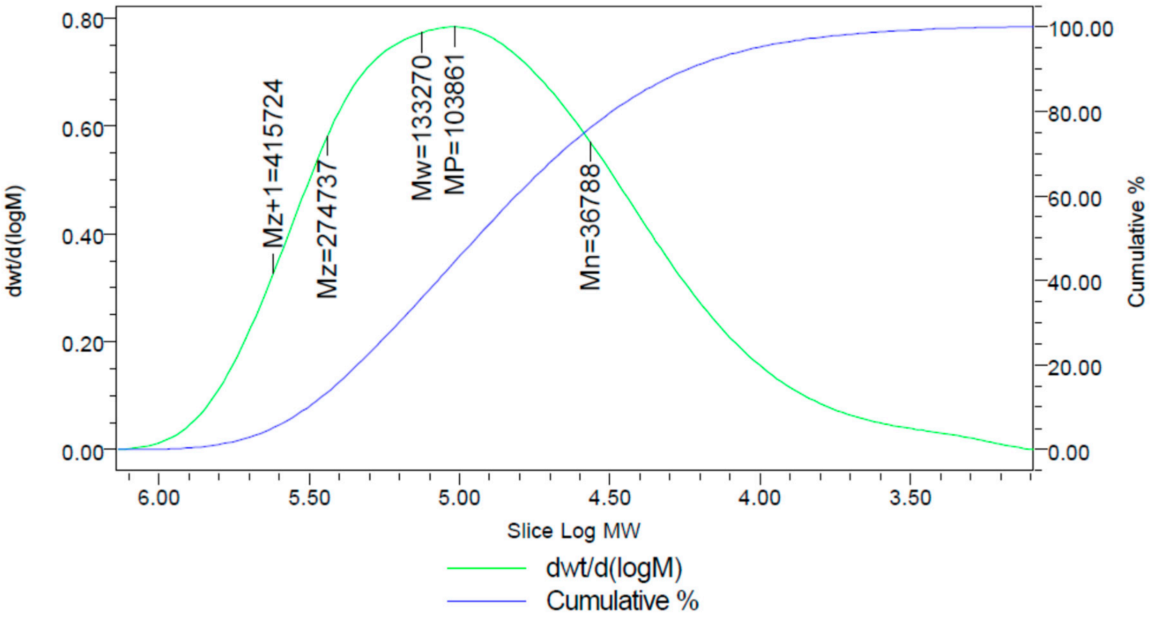

Figure S19. GPC curves and determined molecular weights of ODPA-AFL (two-step, thermal imidization with an azeotropic agent).

GPC Results

	Mn	Mw	MP	Mz	Mz+1	Polydispersity
1	16552	69083	29659	175156	291840	4.173797

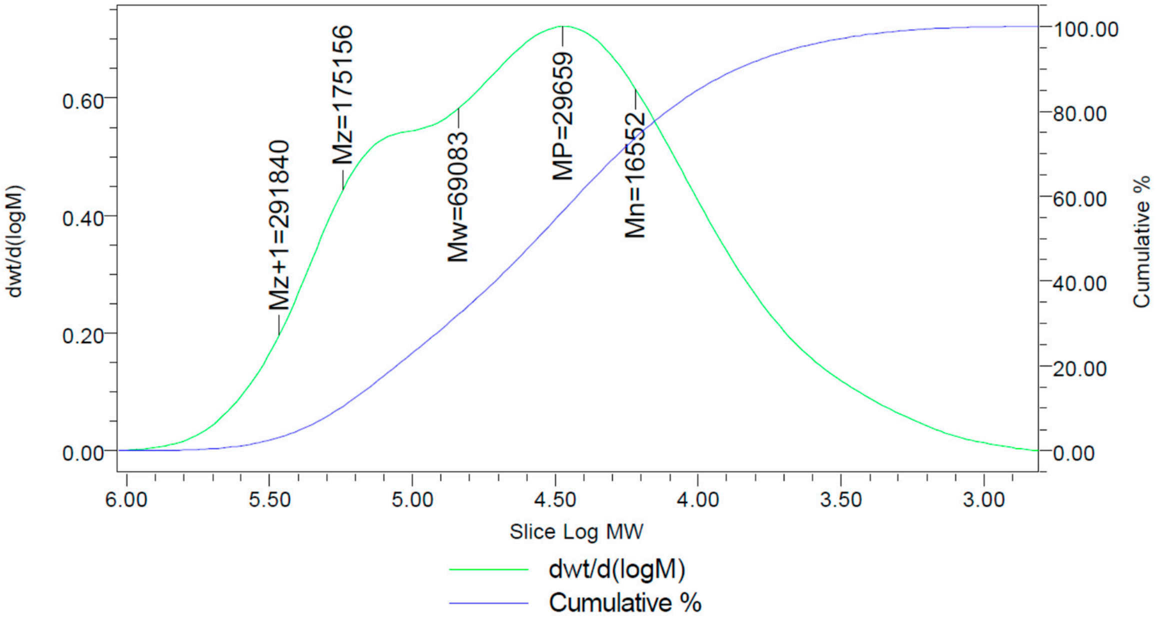

Figure S20. GPC curves and determined molecular weights of **ODPA-AFL** (one-step, NMP).

GPC Results

	Mn	Mw	MP	Mz	Mz+1	Polydispersity
1	253992	461548	426924	692330	935348	1.817175

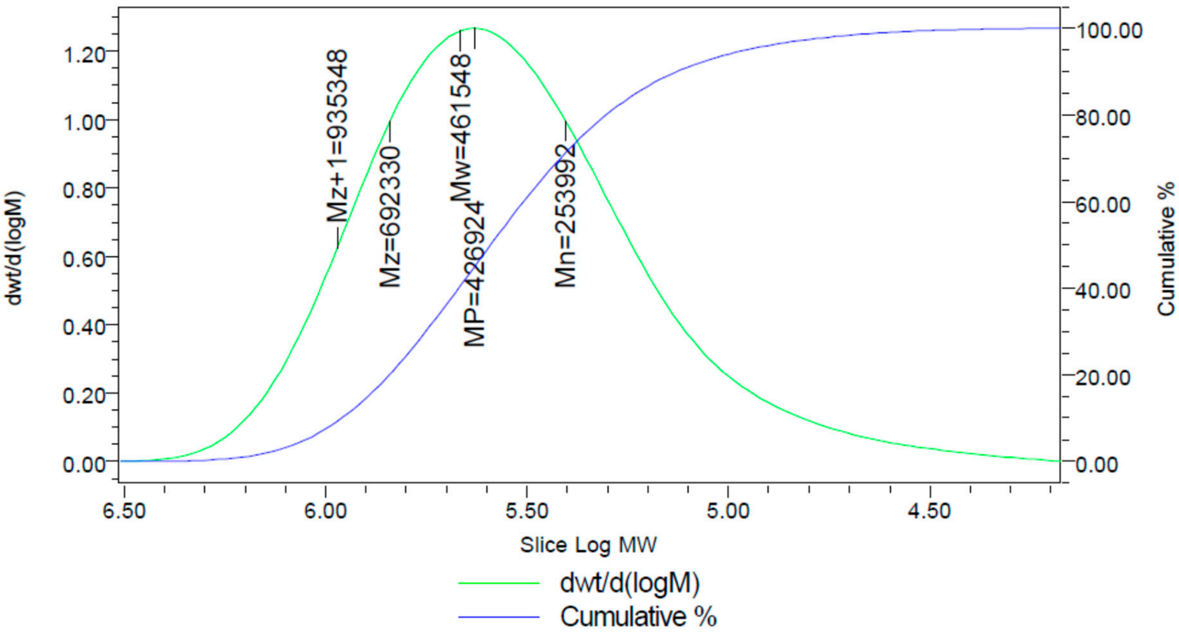

Figure S21. GPC curves and determined molecular weights of ODPA-AFL$_{0.5}$:DABA$_{0.5}$.

GPC Results

	Mn	Mw	MP	Mz	Mz+1	Polydispersity
1	226900	449315	439693	670951	883741	1.980237

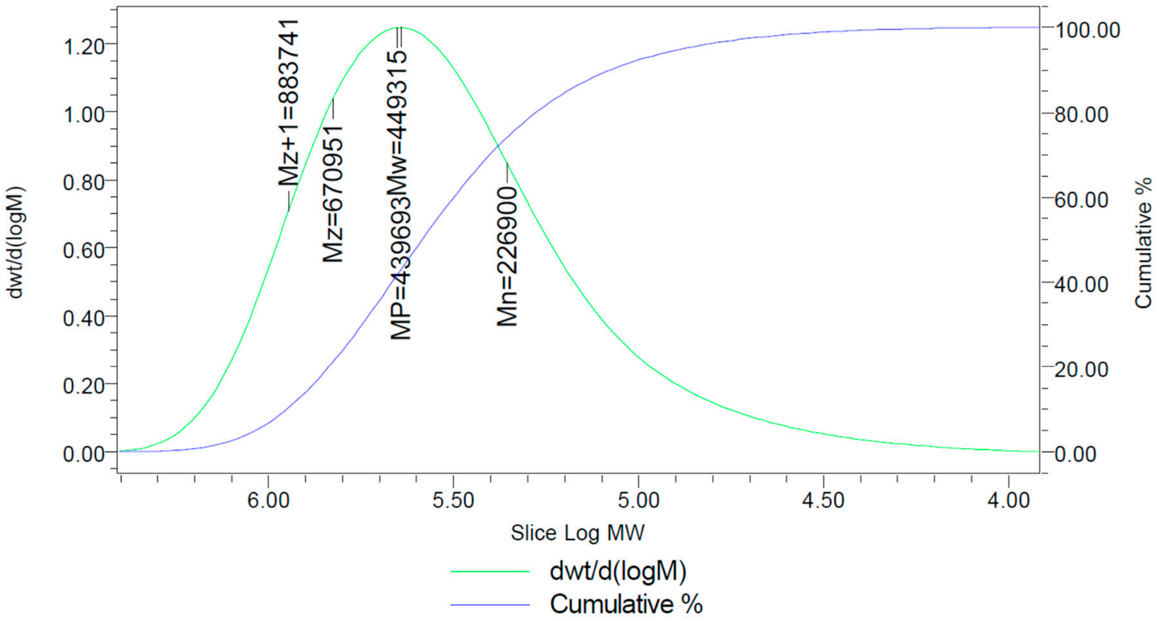

Figure S22. GPC curves and determined molecular weights of ODPA-AFL$_{0.5}$:DABA$_{0.5}$ (one-step, NMP).

GPC Results

	Mn	Mw	MP	Mz	Mz+1	Polydispersity
1	90681	398082	390206	723957	1033133	4.389909

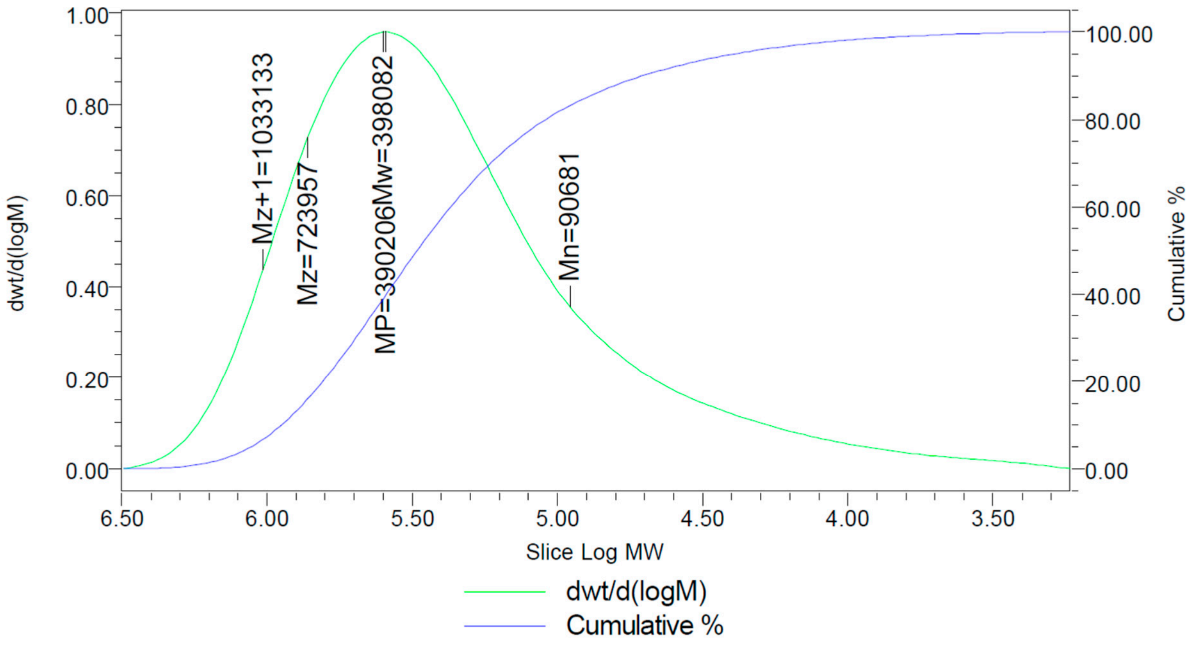

Figure S23. GPC curves and determined molecular weights of BTDA-$AFL_{0.5}$:$DABA_{0.5}$.

GPC Results

	Mn	Mw	MP	Mz	Mz+1	Polydispersity
1	146031	421116	356242	776818	1151612	2.883743

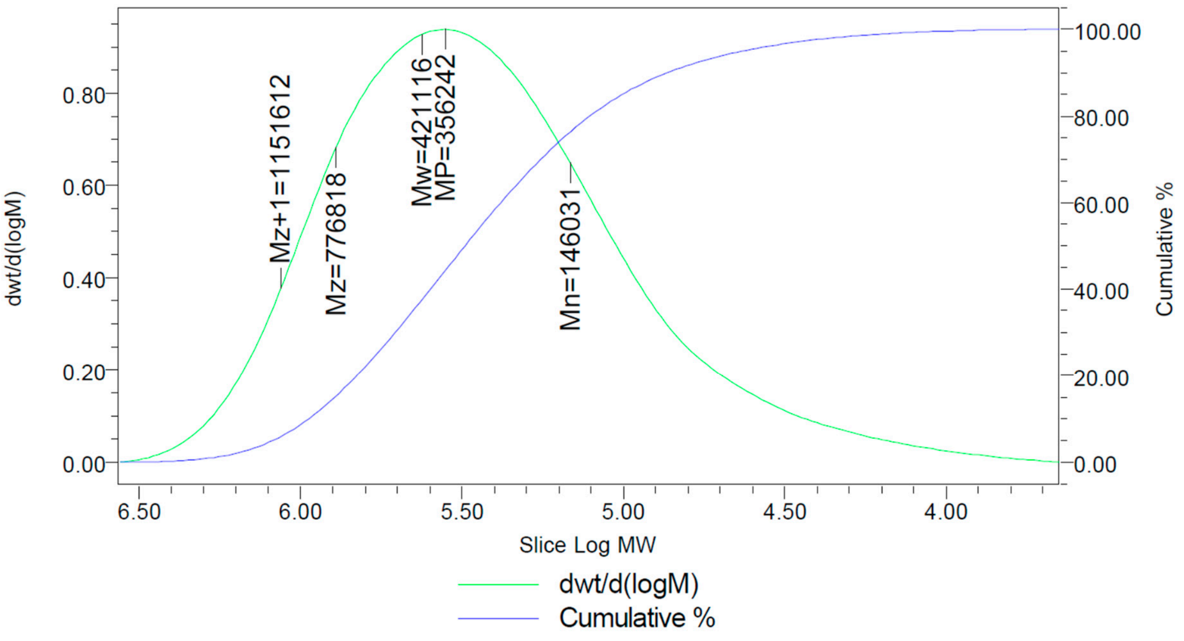

Figure S24. GPC curves and determined molecular weights of BTDA-$AFL_{0.5}$:$DABA_{0.5}$ (one-step, NMP).

GPC Results

	Mn	Mw	MP	Mz	Mz+1	Polydispersity
1	238809	440262	380459	686015	958822	1.843578

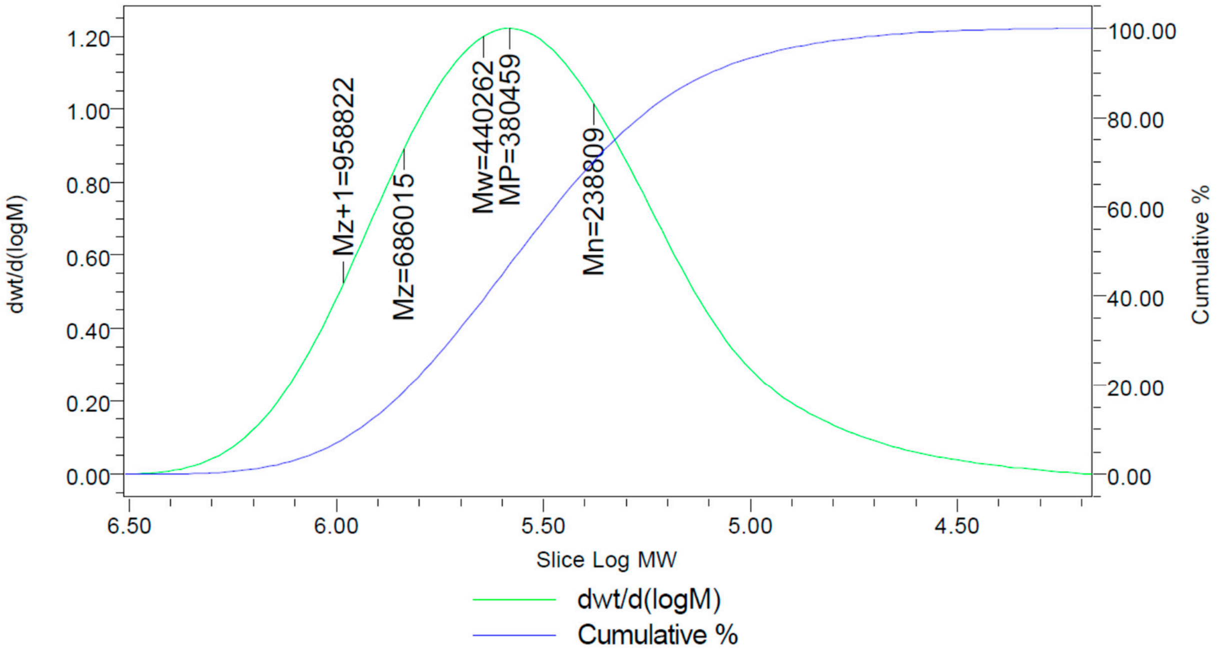

Figure S25. GPC curves and determined molecular weights of BPADA-DABA.

GPC Results

	Mn	Mw	MP	Mz	Mz+1	Polydispersity
1	274912	430395	321133	650902	916768	1.565573

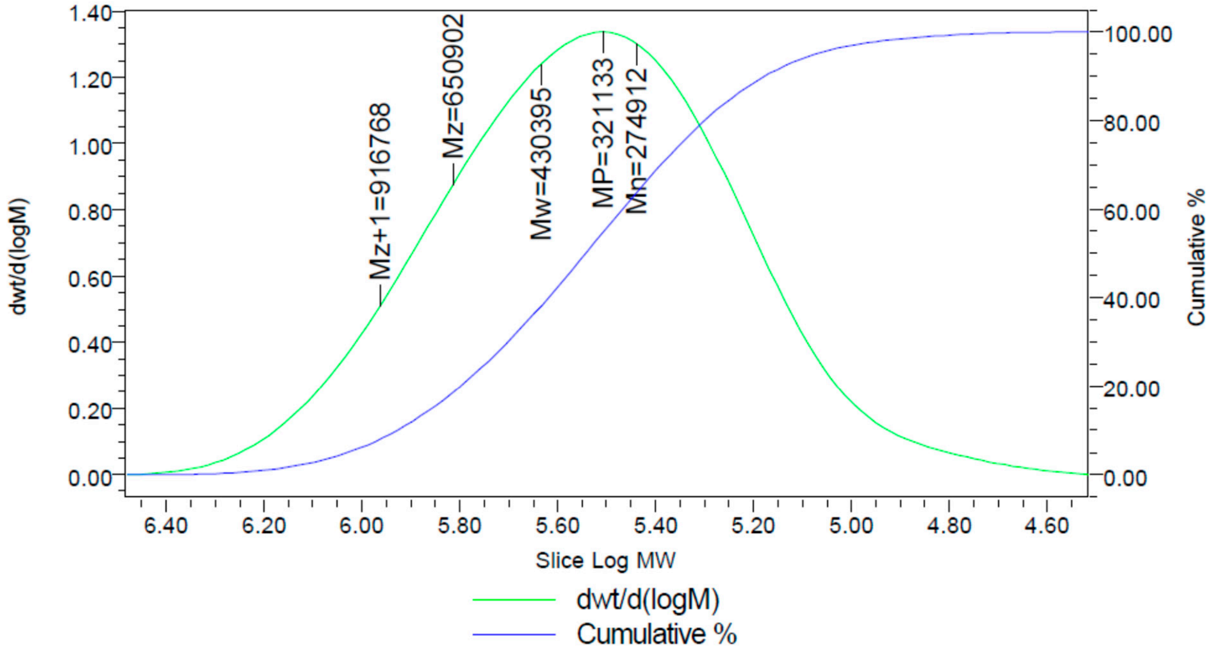

Figure S26. GPC curves and determined molecular weights of 6FDA-DABA.

GPC Results

	Mn	Mw	MP	Mz	Mz+1	Polydispersity
1	345985	537073	426518	794997	1133896	1.552299

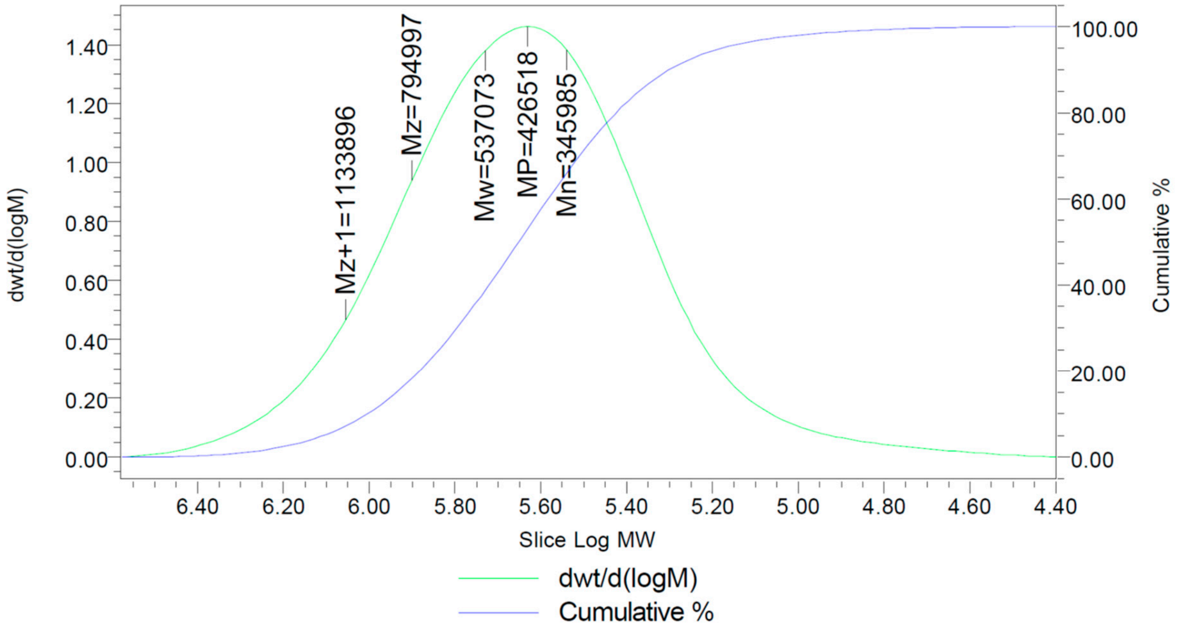

Figure S27. GPC curves and determined molecular weights of 6FDA-DABA (one-step, NMP).

GPC Results

	Mn	Mw	MP	Mz	Mz+1	Polydispersity
1	191022	320480	224274	522483	761142	1.677710

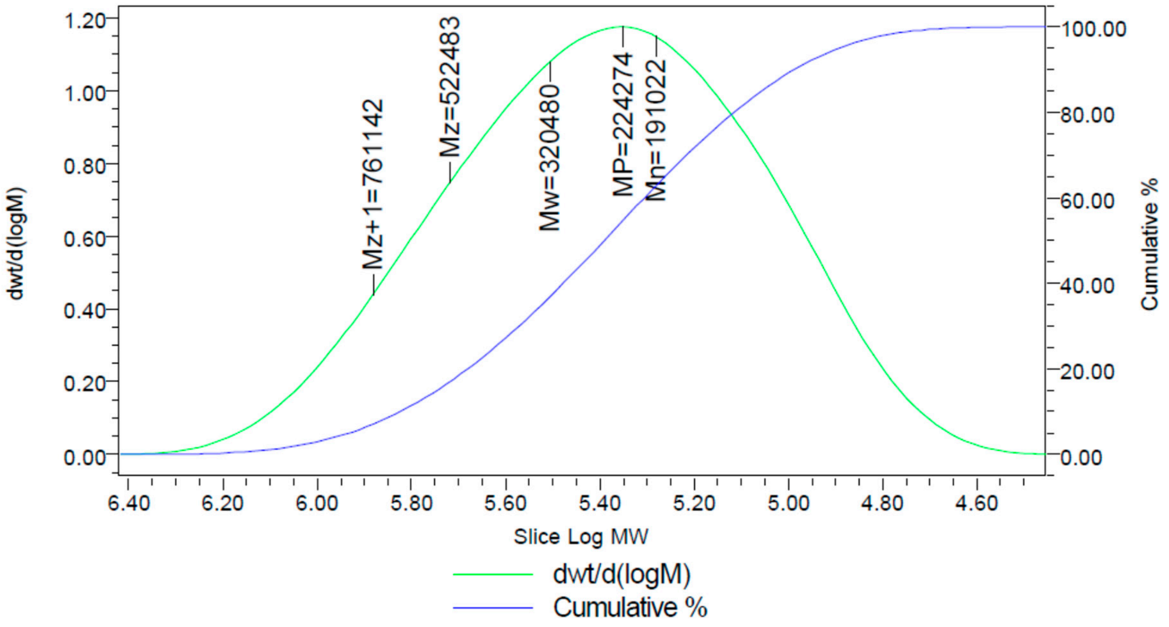

Figure S28. GPC curves and determined molecular weights of PMDA-APH.

GPC Results

	Mn	Mw	MP	Mz	Mz+1	Polydispersity
1						
2	11592	25506	18332	45183	62393	2.200250

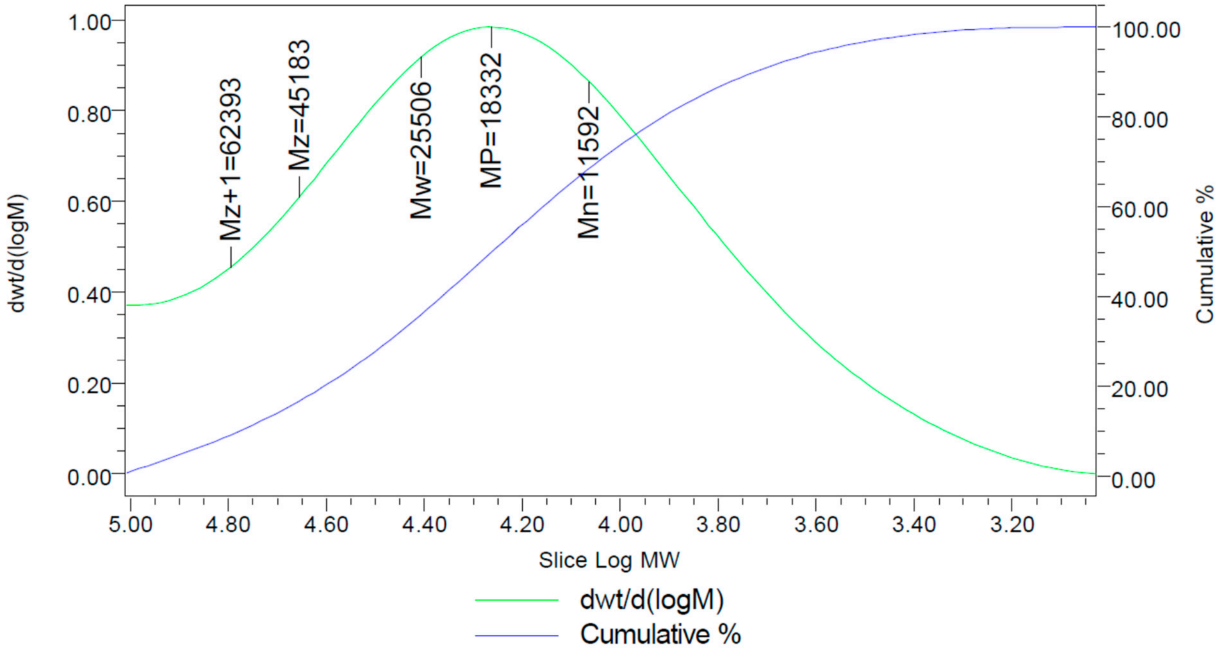

Figure S29. GPC curves and determined molecular weights of NTCDA-APH.

GPC Results

	Mn	Mw	MP	Mz	Mz+1	Polydispersity
1	11609	55704	23240	178547	316109	4.798412

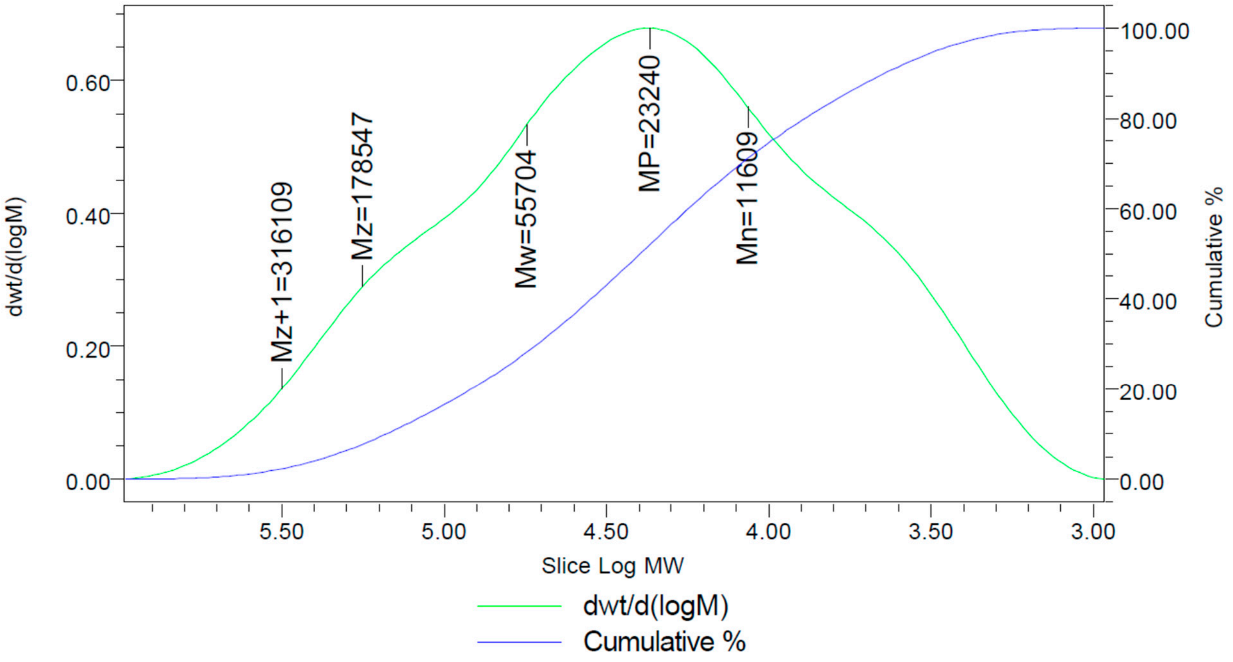

Figure S30. GPC curves and determined molecular weights of NTCDA-$APH_{0.5}$:$DABA_{0.5}$.

GPC Results

	Mn	Mw	MP	Mz	Mz+1	Polydispersity
1	46267	136858	121441	259052	385047	2.958001

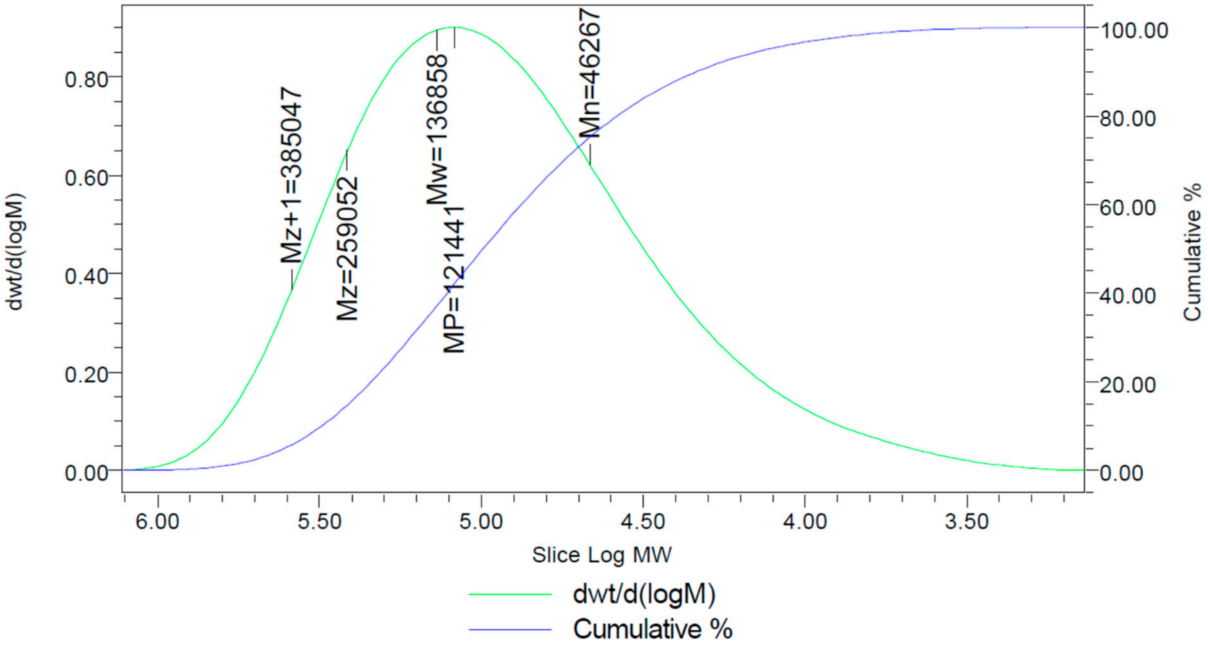

Figure S31. GPC curves and determined molecular weights of TPC-AFL.

GPC Results

	Mn	Mw	MP	Mz	Mz+1	Polydispersity
1	165235	354004	329263	576633	783895	2.142423

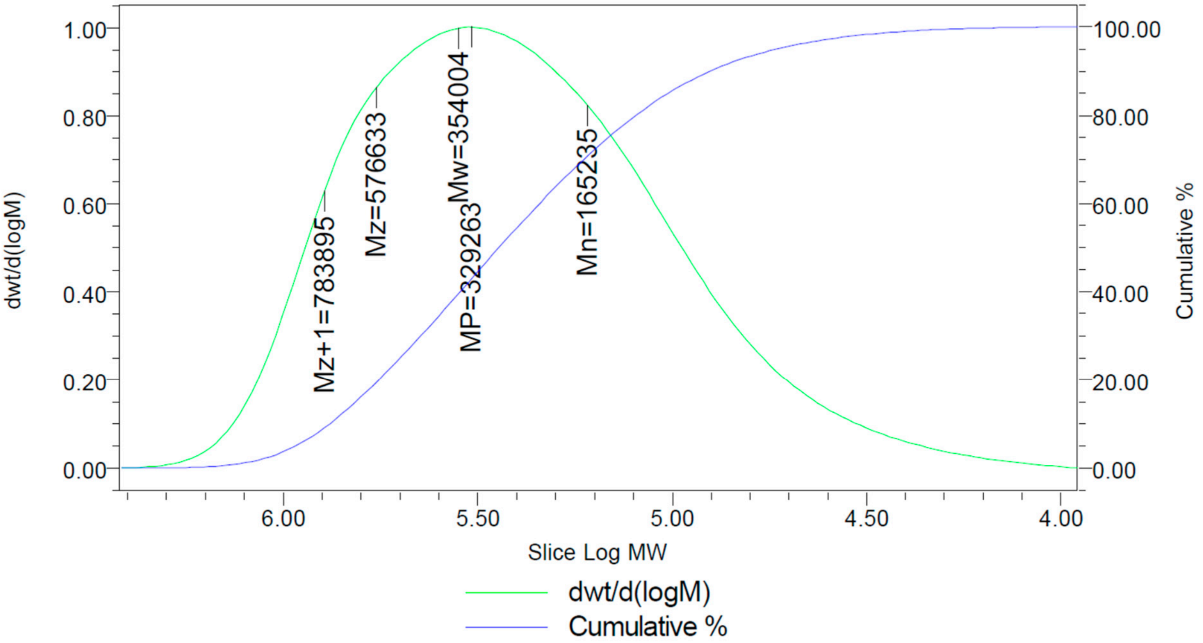

Figure S32. GPC curves and determined molecular weights of TPC-AFL (NMP).

GPC Results

	Mn	Mw	MP	Mz	Mz+1	Polydispersity
1	50940	164008	107740	348239	521539	3.219620

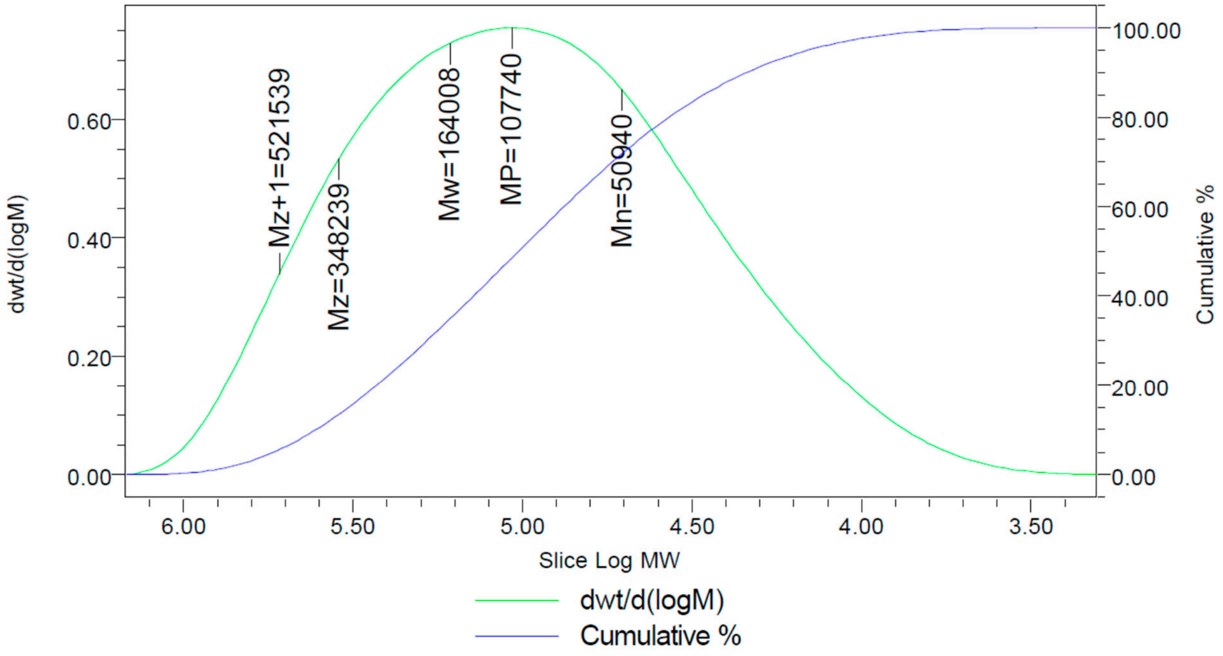

Figure S33. GPC curves and determined molecular weights of TPC-TFMB.

GPC Results

	Mn	Mw	MP	Mz	Mz+1	Polydispersity
1	44042	199332	126660	449378	677356	4.525976

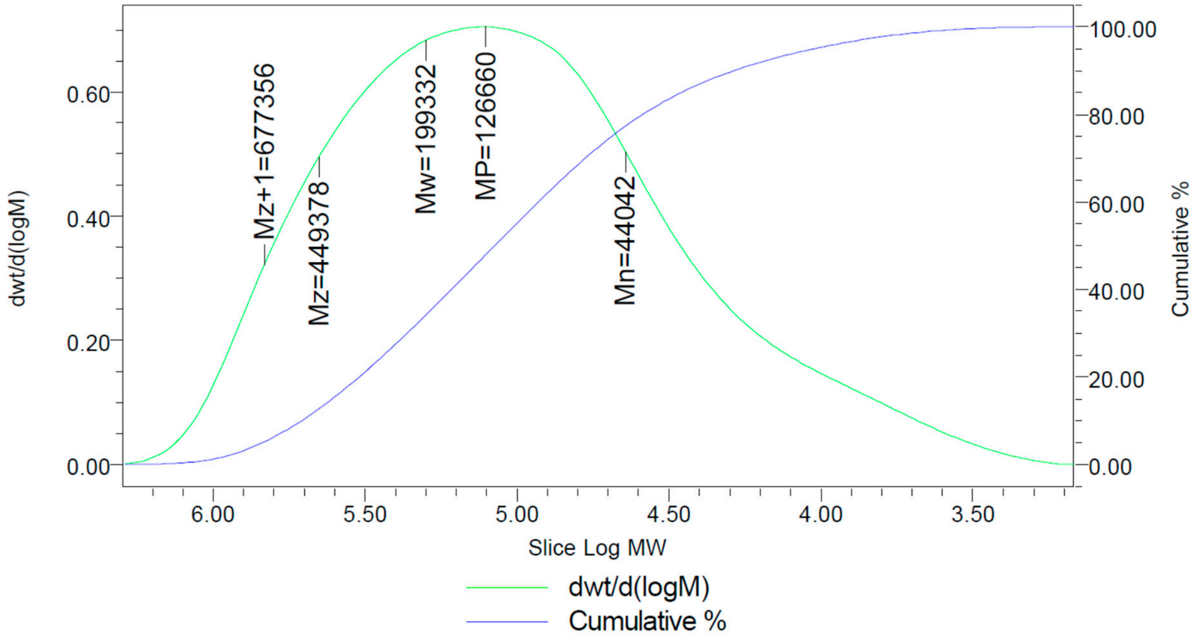

Figure S34. GPC curves and determined molecular weights of TPC-TFMB (NMP).

GPC Results

	Mn	Mw	MP	Mz	Mz+1	Polydispersity
1	57049	213823	222375	400866	573221	3.748074

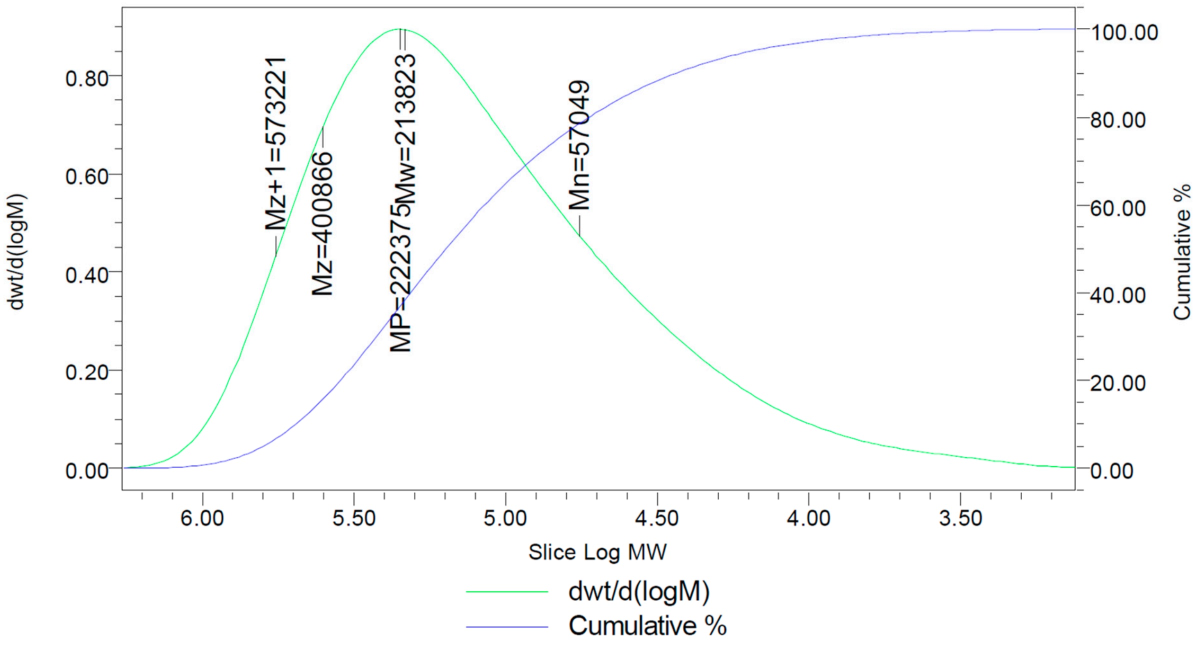

Figure S35. GPC curves and determined molecular weights of ODPA:TPC-AFL.

GPC Results

	Mn	Mw	MP	Mz	Mz+1	Polydispersity
1	91217	250897	251645	428874	593747	2.750539

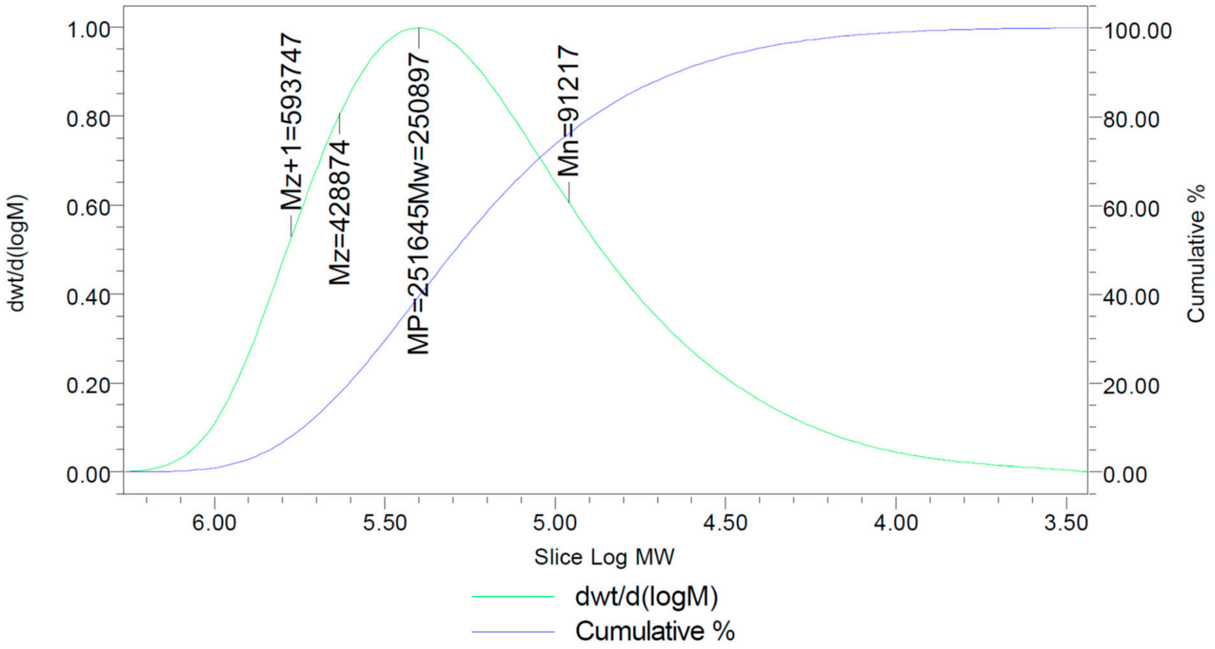

Figure S36. GPC curves and determined molecular weights of ODPA:TPC-AFL (NMP).

GPC Results

	Mn	Mw	MP	Mz	Mz+1	Polydispersity
1	46275	194710	203302	398106	581752	4.207629

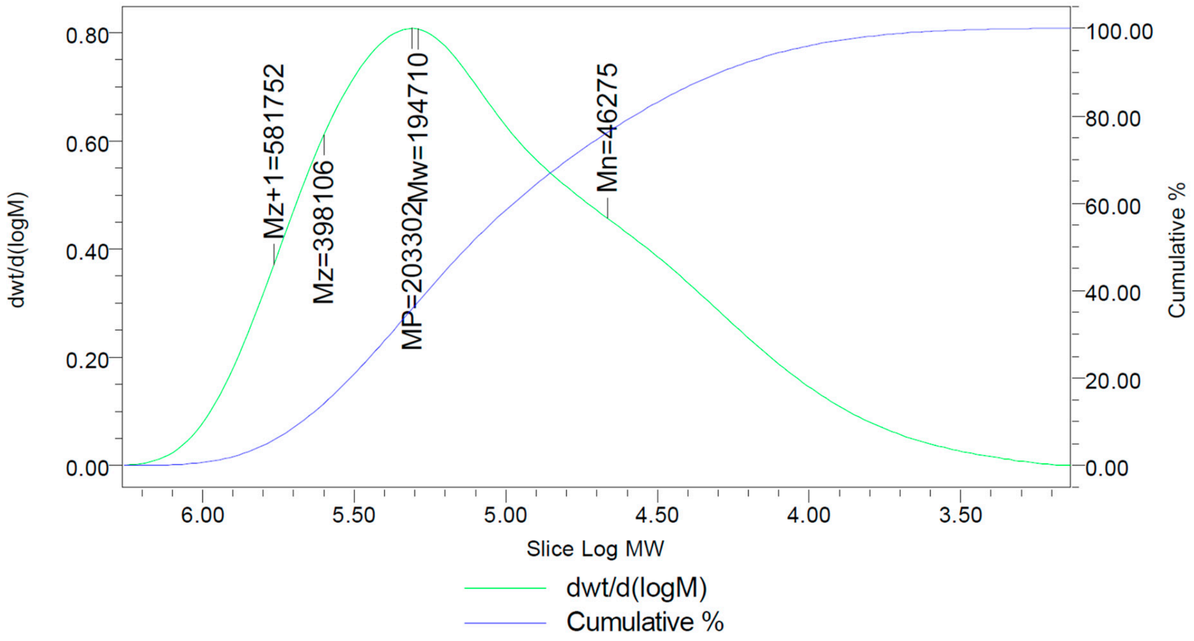

Figure S37. GPC curves and determined molecular weights of BTDA:TPC-AFL.

GPC Results

	Mn	Mw	MP	Mz	Mz+1	Polydispersity
1	30938	129362	154770	295255	455045	4.181372

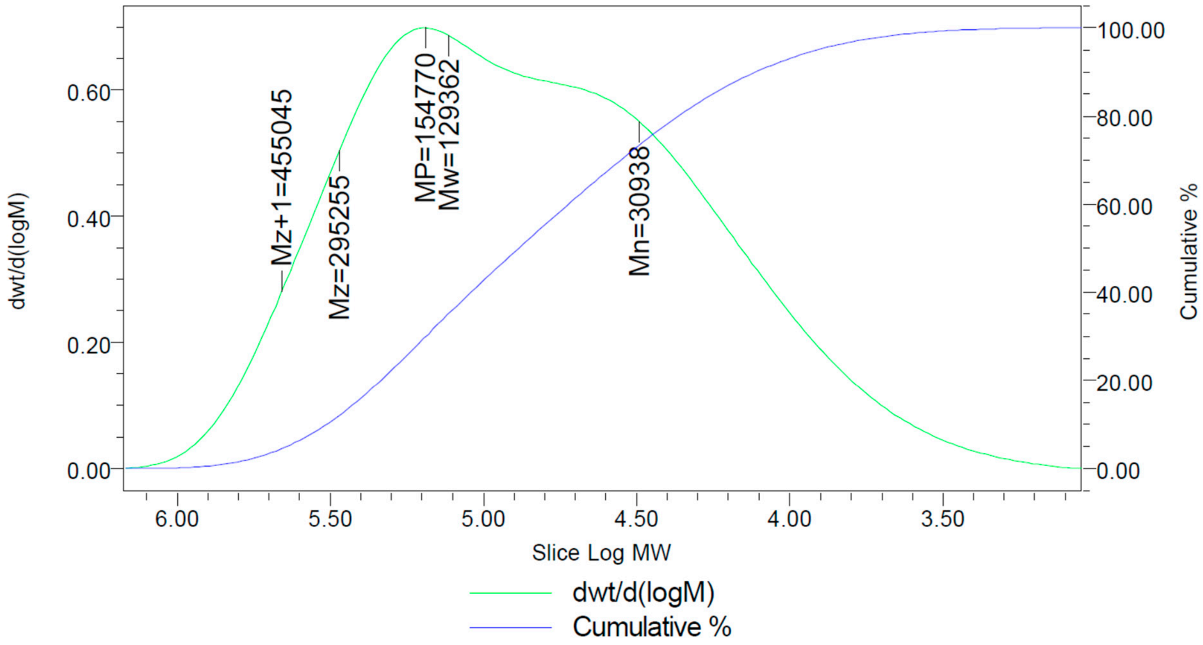

Figure S38. GPC curves and determined molecular weights of BTDA:TPC-AFL (NMP).

GPC Results

	Mn	Mw	MP	Mz	Mz+1	Polydispersity
1	119682	316663	299115	553003	775491	2.645874

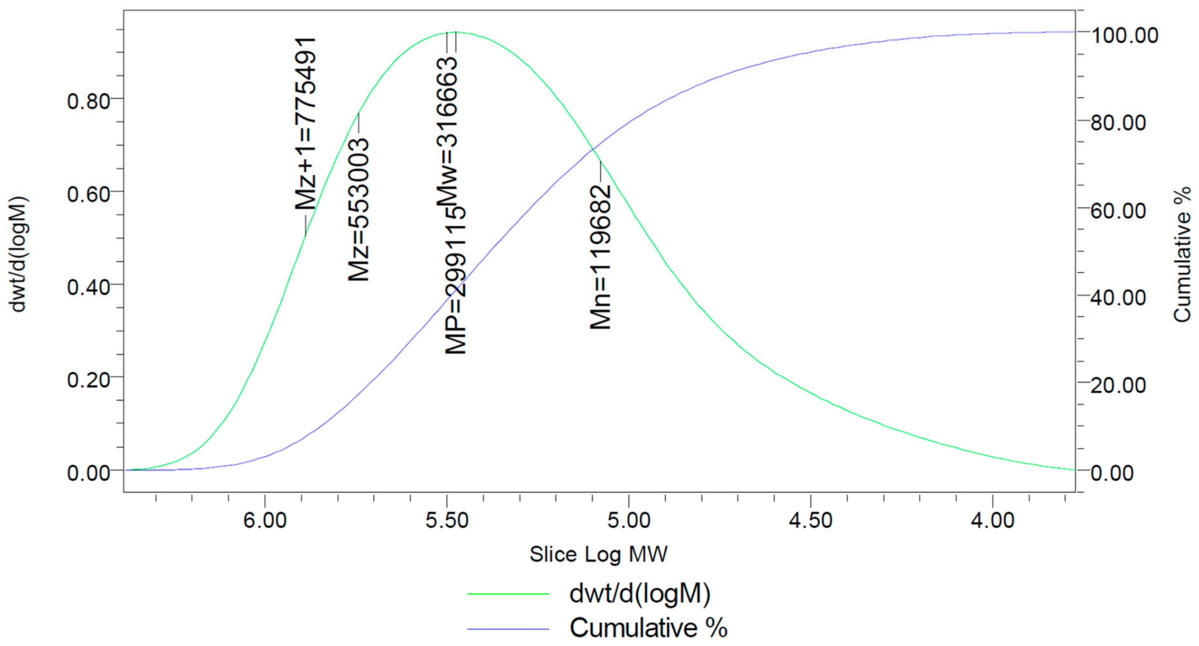

Figure S39. GPC curves and determined molecular weights of ODPA:TPC-TFMB.

GPC Results

	Mn	Mw	MP	Mz	Mz+1	Polydispersity
1	211875	472648	439743	776746	1076543	2.230787

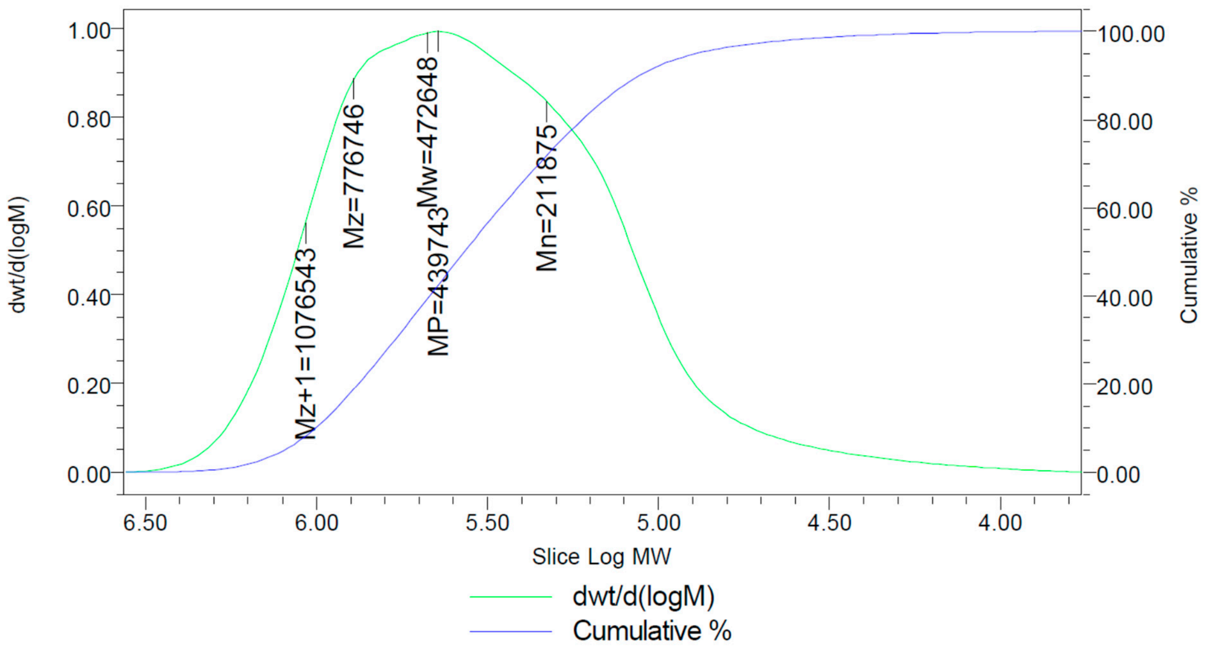

Figure S40. GPC curves and determined molecular weights of ODPA:TPC-TFMB (NMP).

GPC Results

	Mn	Mw	MP	Mz	Mz+1	Polydispersity
1	202021	384747	351176	648234	953586	1.904486

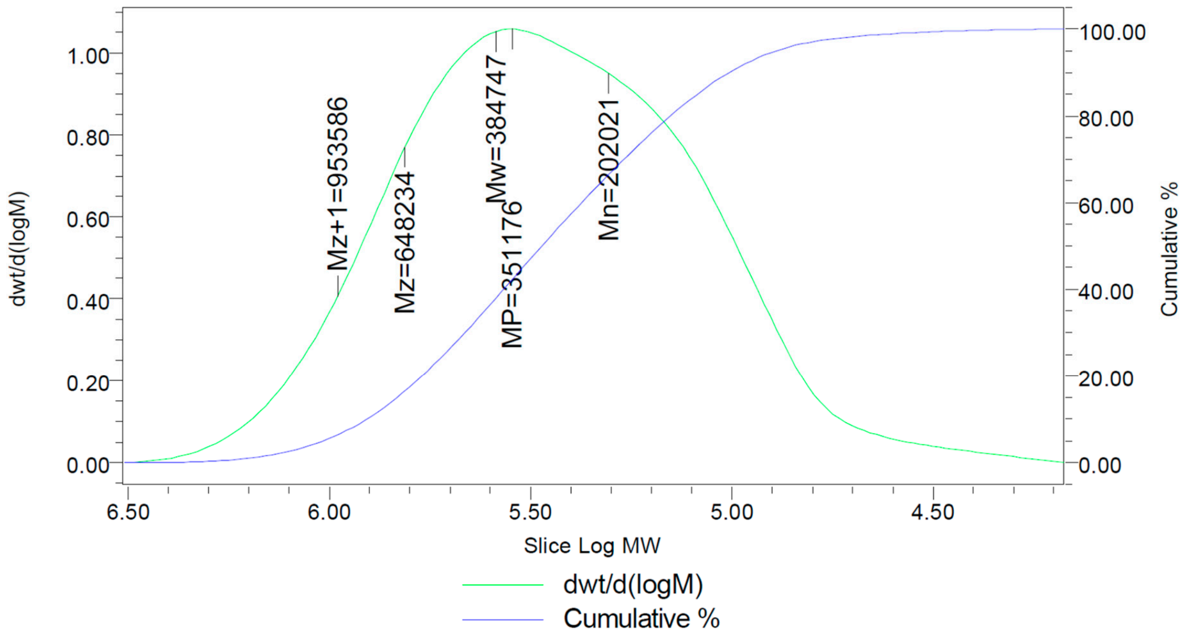

Figure S41. GPC curves and determined molecular weights of BTDA:TPC-TFMB.

GPC Results

	Mn	Mw	MP	Mz	Mz+1	Polydispersity
1	166288	412177	411569	668326	920491	2.478700

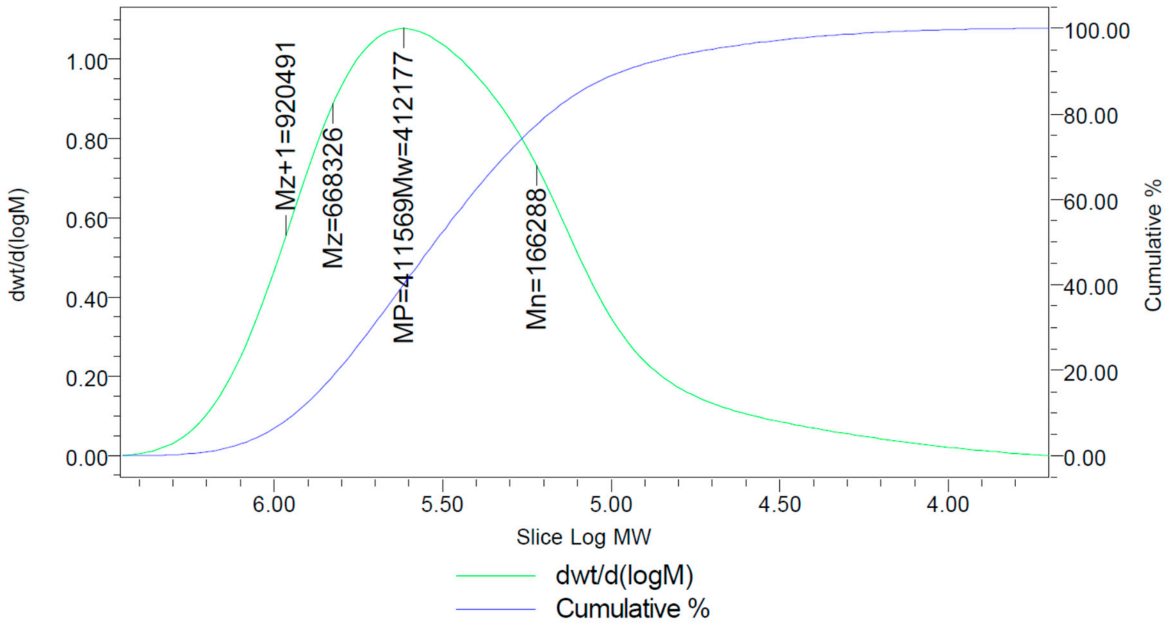

Figure S42. GPC curves and determined molecular weights of BTDA:TPC-TFMB (NMP).
